# Accuracy of Genomic Selection in a Rice Synthetic Population Developed for Recurrent Selection Breeding

**DOI:** 10.1371/journal.pone.0136594

**Published:** 2015-08-27

**Authors:** Cécile Grenier, Tuong-Vi Cao, Yolima Ospina, Constanza Quintero, Marc Henri Châtel, Joe Tohme, Brigitte Courtois, Nourollah Ahmadi

**Affiliations:** 1 CIAT, A.A. 6713, Cali, Colombia; 2 CIRAD, UMR AGAP, F-34398, Montpellier, France; Università Politecnica delle Marche, ITALY

## Abstract

Genomic selection (GS) is a promising strategy for enhancing genetic gain. We investigated the accuracy of genomic estimated breeding values (GEBV) in four inter-related synthetic populations that underwent several cycles of recurrent selection in an upland rice-breeding program. A total of 343 S_2:4_ lines extracted from those populations were phenotyped for flowering time, plant height, grain yield and panicle weight, and genotyped with an average density of one marker per 44.8 kb. The relative effect of the linkage disequilibrium (LD) and minor allele frequency (MAF) thresholds for selecting markers, the relative size of the training population (TP) and of the validation population (VP), the selected trait and the genomic prediction models (frequentist and Bayesian) on the accuracy of GEBVs was investigated in 540 cross validation experiments with 100 replicates. The effect of kinship between the training and validation populations was tested in an additional set of 840 cross validation experiments with a single genomic prediction model. LD was high (average r^2^ = 0.59 at 25 kb) and decreased slowly, distribution of allele frequencies at individual loci was markedly skewed toward unbalanced frequencies (MAF average value 15.2% and median 9.6%), and differentiation between the four synthetic populations was low (F_ST_ ≤0.06). The accuracy of GEBV across all cross validation experiments ranged from 0.12 to 0.54 with an average of 0.30. Significant differences in accuracy were observed among the different levels of each factor investigated. Phenotypic traits had the biggest effect, and the size of the incidence matrix had the smallest. Significant first degree interaction was observed for GEBV accuracy between traits and all the other factors studied, and between prediction models and LD, MAF and composition of the TP. The potential of GS to accelerate genetic gain and breeding options to increase the accuracy of predictions are discussed.

## Introduction

Genomic selection (GS) arose from the combination of new high-throughput marker technologies and new statistical methods that allow the analysis of the genetic architecture of complex traits in the framework of infinitesimal model effects. GS differs significantly from conventional marker assisted selection as it makes it possible to select individuals without prior knowledge of an association between the markers and the trait of interest. GS refers to methods that use genome-wide markers to predict an individual’s genomic value with enough accuracy to allow the selection of individuals based on that prediction alone. GS consists of three steps: (i) inference of the genotype-phenotype relationships in a training population, (ii) prediction of genetic value based on marker genotypes or genomic estimated breeding values (GEBV) in a target population and (iii) selection of individuals based on their GEBV [1]. As GS does not limit the selection to a few markers with a significant association with the trait of interest, it is possible to use markers to select for traits whose genetic control depends on many genes/QTLs with small effects as well as a few genes/QTLs with large effects. GS is thus perfectly suited for breeding highly polygenic traits, such as yield, drought tolerance, and resource use efficiency [1,2].

Several statistical prediction models have been developed that differ in the assumptions they make about the effects of markers and the variance of such effects across the genome: random effects drawn from a normal distribution with equal variance for all markers (ridge regression best linear unbiased prediction), random effects drawn for each marker from a normal distribution with its own variance (Bayes A), or the probability that the marker has no effect at all (Bayes B). Semi-parametric and non-parametric regressions have been developed to map genotypes to phenotypes for traits with non-additive genetic architecture [3]. However there is no single best model and the accuracy of the different models depends on the characteristics of the target population (effective population size, linkage disequilibrium, population structure, etc.) and the traits targeted (heritability, number of QTLs and the size of their effects, and the relative magnitude of additive and non-additive genetic variance).

To date, research on GS has mainly focussed on livestock breeding. Its use in plant breeding schemes began [4] with investigations of the accuracy of GEBV predictions relying on simulation studies [5–7]. The first GS studies in crops using experimental data were based on populations generated from biparental crosses of maize [8,9] and wheat [10] before shifting to populations with more a complex genetic structure such as diversity panels of wheat [7], maize [11], and oats [12], and advance breeding lines derived from multiple crosses in wheat [10], or from a nested association mapping populations in maize [13].

GS is rather new in rice (*Oryza sativa*). A simulation study comparing the accuracy of nine GS methods in predicting eight traits in a collection of 110 Asian cultivars [14] concluded that accuracy depended to a great extent on the traits targeted and that reliability was low when only a small number of cultivars was used for validation. Two studies reported on the application of GS to empirical data on rice. Based on a set of 413 highly diverse accessions with strong population structure, two separate studies [15,16] revealed that the most accurate predictions can be obtained through stratified sampling of the training set. More recently, genomic predictions based on a population of 383 elite breeding lines from the International Rice Research Institute’s (IRRI) irrigated rice breeding program, and 73,147 markers concluded that one marker every 0.2 cM is sufficient [17]. GS in rice was shown to better capture the genetic variance of small-effect QTLs that cannot be detected by genome wide association studies GWAS [16]. The authors showed that the proportion of phenotypic variation explained by all QTLs identified by GWAS on the same population [18] was lower than the proportion obtained with a model based on all markers, i.e a potential 65% genetic gain.

Since 1992, CIAT (International center for tropical agriculture) and Cirad (French agricultural research and international cooperation organization for sustainable development) have developed a rice breeding program for Latin American and the Caribbean based on the improvement of synthetic populations through recurrent selection (RS) [19,20]. The program started with the development of a base population P_0_ through inter-crossing of approximately 60 complementary founder accessions. The resulting base population was recombined several times to generate a large number of recombinants, leading to a synthetic population of rather low linkage disequilibrium (LD) endowed with sufficient genetic variability for future selection. The RS scheme includes three stages conducted recurrently: (i) evaluation of individuals derived from a population P_n_, (ii) selection with mild pressure of the best individuals to gradually increase the frequency of favourable alleles at loci involved in complex traits (additive and epistasis effects) [21], (iii) inter-crossing of the selected individuals to form a new population P_n+1_ with an improved mean. This scheme is facilitated by the presence of a recessive nuclear male-sterility (*ms-IR36*) gene located on chromosome 2, segregating within the population [22]. At the end of each cycle, the selected best individuals are also used for the development of improved varieties through conventional pedigree breeding. Based on the assumption that the RS population breeding scheme provides a favourable framework for the application of the GS approach, we started investigating the accuracy of GEBV prediction between two successive generations of RS as well as the sensitivity of the accuracy to different population parameters and prediction methods.

Here, we present the results of a large array of cross validation experiments exploring the effects of the size of the training population, the density of markers in relation with linkage disequilibrium (LD) and minor allele frequency (MAF), the relatedness between the training and the breeding population, the heritability of the target traits and the type of prediction model on the accuracy of GEBV prediction using phenotypic and genotypic data on 343 S_2:4_ lines extracted from our upland rice synthetic population.

## Materials and Methods

### Plant material

Numerous populations have been developed by Cirad and CIAT for the purpose of breeding upland rice varieties for Latin America and the Caribbean. Four populations that underwent more than 10 cycles of RS with very mild selection for yield under favourable upland conditions form the base of the CIAT upland rice-breeding program. To test the effect of structure in GS, the four populations were combined to build a training population (TP). One hundred S_0_ plants with a heterozygous genotype at the male sterility locus *ms-IR36* [22] were extracted from each of the four synthetic populations PCT4-C0, PCT4-C1, PCT4-C2 and PCT11-C1. PCT4-C0 was created in 1995 by crossing seven elite lines from the CIAT, IRRI and Embrapa upland rice program with a source of sterility originating from a base population (CNA-IRAT A) comprising 40 founder accessions. PCT4-C1 is the product of several cycles of RS on PCT4-C0, with very mild selection for yield under favourable upland savannah rice growing conditions in Colombia. PCT4-C2 is the product of several cycles of RS on PCT4-C0, plus not well-documented enrichments in Bolivia. PCT11-C1 was created in 1996 by crossing 17 accessions of diverse origins with representatives of PCT4-C0 population bearing the [ms ms] genotype and was improved through RS breeding in Bolivia [19]. The 400 S_0_ were advanced to S_1:2_ through single seed descent while marker assisted selection was performed at the *ms-IR36* locus. For each S_1:2_ one homozygote fertile plant was advanced to S_2:3_ for DNA extraction and to S_2:4_ for phenotyping, using a bulk breeding procedure.

### Target traits and phenotypic data

The phenotyping experiment took place in the Colombia Agricultural Research Corporation (Spanish acronym CORPOICA) Center (La Libertad) located in Villavicencio, Meta, Colombia (9°6’N; 73°34’W; 330 m asl.) during the 2013 rainy season. No specific permission was required for phenotyping at the CORPOICA facility as an existing agreement links CIAT and CORPOICA to carry-on field trials at the La Libertad location (convenio de accuerdo CIAT-CORPOICA # 20130181 del 15 de Mayo de 2013). Furthermore, the field studies did not involve endangered or protected species. The field used for evaluation is typical of the savannah conditions, with acid soils (pH < 5.0) and high aluminium content (> 75 ppm). The field evaluation was conducted under standard agricultural practices. The experimental design was an alpha lattice with 21 blocks of 17 plots for each of the two complete replicates. It comprised 346 S_2:4_ lines (86, 83, 84 and 93 lines from the three PCT4 and PCT11-C1 populations) with three standard checks (Oryzica Sabana 6, Oryzica Sabana 10, Cirad 409) and 10 additional entries to complete the design. The experiment began with dry direct seeding. Each experimental plot comprised two 3 m-long rows with 26 cm between row spacing. Seed density was one gram of seed per meter.

The target traits were days to flowering (FL), plant height at maturity (PH), weight of an individual panicle (PW) and grain yield (YLD), assumed to represent together a wide range of heritability and genetic architecture. FL was recorded as the date when 50% of the plants in the plot had flowered. PH was the average height (cm) of five plants in the plot. Five panicles were collected along a distance of one meter in the row, weighed and averaged to obtain the individual PW (g). Grains from these five panicles were added to the grains collected from plants along the remainder of the one-meter length to give YLD expressed in g/m.

First, a diagnostic module was run using the “influence” option to detect outliers among the individual observations. It included the “iter =“ sub-option that updates both fixed effects and covariance parameters by refitting the mixed model when an observation is deleted. (http://support.sas.com/documentation/cdl/en/statug/63962/HTML/default/viewer.htm#statug_mixed_sect027.htm). This diagnostic procedure led to the elimination of two elementary data. The selection of entries with both phenotypic data and genotypic data lead to a final number of 343 S_2:4_ lines, which were used to develop the prediction model.

For each trait, experimental data were analyzed using the SAS 9.3 MIXED procedure [23] to estimate the BLUP values and heritability. The ANOVA model was Y_ijk_ = μ + g_i_ + R_j_ + b_k(j)_ + e_ijk_ in which Y_ijk_ was the phenotype, μ the overall mean, g_i_ was the genotype effect considered as random, R_j_ was the replicate effect considered as fixed, b_k(j)_ was the random effect of the block within a replicate, and e_ijk_ was the residual considered as a random effect.

Variance components were obtained, and a BLUP value was extracted for each line to be used in the GS models. The phenotypic BLUP x accession matrix is available for download at http://tropgenedb.cirad.fr/tropgene/JSP/interface.jsp?module=RICE as "SEPANG dataset".

For each trait, narrow sense heritability was calculated using the ratio $h_{ns}^{2}=\frac{\sigma_{S_{2:4}}^{2}}{\sigma_{P}^{2}}$, where $\sigma_{S_{2:4}}^{2}$ is the genotypic variance obtained from the experimental data (assuming only additive genetic variance among S_2:4_ families) and the phenotypic variance $\sigma_{P}^{2}=\sigma_{S_{2:4}}^{2}+\sigma_{e}^{2}$, where $\sigma_{e}^{2}$ is the residual variance obtained from the ANOVA.

Multi-dimensional analysis of phenotypic data by factorial discriminant analysis (FDA) was performed and pairwise Fisher distance between subpopulations was calculated using the XLSTAT package [24].

### Genotypic data

Genomic DNA was extracted from bulked leaf tissue from 15 S_2:3_ plants following the MATAB method described in [25] and diluted to 100 ng/μl. Genotyping was done at Diversity Arrays Technology Pty Ltd (DArT P/L), Australia, following a method combining Diversity Arrays Technology (DArT) and next generation sequencing DArTseq described in Courtois et al. [26]. The proprietary analytical pipeline developed by DArT P/L was used to produce DArT score tables and SNP tables. It provided 9,681 SNP markers of which 8,385 had less than 20% missing data for 343 S_2:3_ lines. The average rate of missing data among the 8,385 SNP was 3.2%. Genotypic profiles for these missing data were estimated using Beagle v3.3 through the open source Synbreed package available from the R website (http://cran.r-project.org/web/packages/synbreed/index.html). Beagle uses a hidden Markov model to infer haplotypes and imputes sporadic missing data on large scale phase known or unknown genotype data sets [27]. The imputation procedure was performed on markers with less than 20% missing data and an MAF >5% and resulted in complete dataset for 8,336 SNP markers with imputed genotypes selected with a >0.75 probability. The 8,336 SNP x accession matrix is available for download at http://tropgenedb.cirad.fr/tropgene/JSP/interface.jsp?module=RICE as "SEPANG dataset".

### Methods for characterizing the population

#### Population structure

The structuring of the 343 S_2:3_ lines into four subpopulations corresponding to PCT4-C0, PCT4-C1, PCT4-C2 and PCT11-C1 was investigated by FDA [24]. The analysis was performed with the coordinates of the S_2:3_ lines on the 10 first axes of a principal components analysis, using the genotype on the 3,675 SNP loci with no missing data.

#### Genetic differentiation

Genetic variation between subpopulations was estimated using a pairwise *F*_*ST*_ test that estimates genetic differentiation based on allele frequency [28]. The *F*_*ST*_ statistics were calculated over the genome using 3,675 markers with no missing data with Arlequin software [29].

#### Effective population size (Ne)

*Ne* was estimated for each of the four subpopulations as well as for the whole population, from genotypes at 3,675 SNP loci. *Ne* was calculated using the linkage disequilibrium method of Waples and Do [30], re-implemented in NeEstimator V2.01 [31].

#### Kinship among the 343 S_2:3_ lines

A marker-based kinship matrix was generated using the 8,336 SNP available after imputation. Kinship estimates were obtained by calculating a distance matrix (d_*ij*_ = *n*/8,336; *n* being the number of SNPs that differ between line *i* and line *j*), which was converted into a similarity matrix by subtracting all values from 2, then scaled to rank values from 0 to 2, using the Synbreed package [32].

#### Pairwise linkage disequilibrium

LD within the panel of 343 S_2:3_ lines was evaluated for each chromosome by computing r^2^ between all pairs of SNP markers of a given chromosome, as defined by Hill and Roberson [33]. The r^2^ were computed using the Synbreed package, which called for a R function under the option pairwiseLD use.plink = FALSE [32].

### Population parameters considered for their effect on the accuracy of predictions

#### Size of the training population

The effect of the size of the training population (TP) was analyzed by varying the fraction (k) of the population used for the model calibration with respect to the fraction used for model validation (VP). Three fractions were considered, k = 3, 6 and 9, where 1/k of the total population (343 S_2:4_ lines) was used for the validation of a prediction model developed on (k-1)/k of the population.

For the main cross validation experiment, incidence matrices were built to evaluate the effects of MAF, LD and the resulting number of markers. Nine sets of SNP markers were selected by combining three threshold values of MAF (≥ 2.5, ≥ 5 and ≥ 10%) with three threshold values of LD (r^2^ ≤ 0.75, r^2^ ≤ 0.90 and r^2^ ≤ 1). Markers were first retained on the basis of MAF threshold and second on the basis of pairwise r^2^ values with all other markers on the carrier chromosome. MAF threshold values were selected to assess the impact of including less frequent alleles in the prediction model, while the LD threshold values were selected to assess the impact of redundancy caused by linked loci.

In a supplementary set of cross validation experiments, the effects of LD and MAF on the accuracy of predictions were further analyzed by combining five threshold values of MAF (≥ 0.01, ≥ 2.5, ≥ 5, ≥ 7.5 and ≥ 10%) with seven threshold values of LD (r^2^ ≤ 0.4, ≤ 0.5, ≤ 0.6, ≤ 0.7, ≤ 0.8, ≤ 0.9 and ≤ 1). The 35 sets of SNP markers were used for cross validation with the ridge regression (RR-BLUP) prediction method (see below) with k = 3-fold cross validation, for each of the four traits considered.

#### Random sampling of markers

Random sampling was performed to distinguish the specific effect of a particular combination of MAF and LD on the accuracy of prediction from the effect of an equivalent number of randomly chosen markers. Ten incidence matrices were sampled, from 100 SNPs to 7,200 SNPs with an increment step of 100, resulting in 720 matrices. These matrices were used with the k = 3-fold cross validation experiment with RR-BLUP prediction method for each of the four traits.

#### Relationship between the training and the validation populations

The effect of varying degrees of relationship between the TP and VP was investigated in a set of cross validation experiments by comparing the accuracies of prediction obtained under three strategies for the assignment of the S_2:4_ lines to the TP and VP populations: (i) random assignment, where the 343 S_2:4_ lines were considered as being derived from a single population, i.e. 2/3 being randomly assigned to the TP and 1/3 to the VP; (ii) balanced assignment based on the stratification of the 343 S_2:4_ lines into four subpopulations (PCT4-C0, PCT4-C1, PCT4-C2 and PCT11-C1), where each subpopulation contributed 2/3 to the TP and 1/3 to the VP; (iii) unbalanced assignment where, in turn, all lines from three subpopulations were used as TP and all lines from the subpopulation that was left out were used as the VP. These six case studies were tested for accuracy using the RR-BLUP method for the four traits and 35 incidence matrices.

### Methods for predicting GEBV among S_2:4_ lines

As a first step in exploring the potential of genomic selection in our rice population breeding program, we assumed a strictly additive genetic model among the S_2:4_ families. This was a reasonable approximation in the context of population improvement with mild selection pressure for the traits of interest. Parametric whole genome regression methods are widely used to handle these additive genetic models. These methods are based on well-established theories and to some extent apply the regularization technique to resolve the p >> n problem. The parametric whole genome regression methods differ considerably in the way the regularization parameter λ is obtained and used in calculations [34]. Several user-friendly computing tools have been developed in the R environment (http://www.r-project.org/) to perform whole genome regression.

Five methods were applied (three frequentist and two Bayesian) to test the different hypotheses concerning the marker effects. The frequentist methods were the genomic best linear unbiased prediction (G-BLUP), the ridge regression best linear unbiased prediction (RR-BLUP), and the least absolute shrinkage and selection operator (LASSO). The Bayesian methods were Bayesian ridge regression (BRR) and Bayesian LASSO (BL). In G-BLUP, the first step consisted in using the incidence matrix X linking markers to individuals to estimate the genomic relationship matrix (G), a matrix of covariance between individuals based on observed similarity at the level of the markers [35]. A mixed model was implemented using the R-ASReml package [36] using the G relationship matrix to produce the predictions for each individual (i.e. individual BLUP). RR-BLUP [37] is another early but still widely used method for genomic prediction [9,38]. The method consists in estimating simultaneously the effect of all markers listed in X, through an optimization function that minimizes both the sum of squared errors and the sum of squared marker effects multiplied by the λ parameter. This method gives similar individual predictions to those produced by G-BLUP [39]. By shrinking all marker effects with the same degree and including all markers in the model, both RR-BLUP and G-BLUP methods imply that the trait is controlled by many loci with small effects. RR-BLUP was implemented using the rrBLUP package [40]. The LASSO regression method drives some marker effects to exactly zero, selecting at most the same number of predictors as the number of observations [41]. When predictors are highly collinear, LASSO retains only one random marker per group of correlated predictors, which could be non-physically linked to underlying genes controlling trait variation. LASSO retains or discards markers, which often results in high variance and high prediction error by the full model. A parameter λ that minimizes the prediction error was found by cross validation and used to predict GEBVs in the VP. We used the Glmnet package [42] to implement the LASSO method. Bayesian methods are used to select variables and to shrink estimates [34]. Using this approach, a prior was assigned to all model unknowns, while the intercept and the vector of fixed effects were assigned flat priors. Under a Bayesian model, a separate variance was estimated for each marker, and the variance was expected to follow a specified prior distribution [43]. The hyper-parameter λ was formulated using a Gamma distribution to match the expected proportion of variance accounted for by the corresponding element of the linear predictor [44]. The rate and shape parameters of the Gamma distribution assigned to the regularization parameters were defined following the guidelines presented by de los Campos [45]. The BRR and BL regression models used in this study considered marker-homogeneous and marker-specific shrinkage of estimates effects, respectively. For both methods, the estimate of variances effects was calculated through a Gibbs sampling procedure based on the posterior distribution conditional on all other effects. The BL led to stronger shrinkage of estimates of marker effects toward zero for markers with small effects and less shrinkage of estimates for markers with sizable effects than BRR. The BL method on additive SNP effects was performed as proposed by Park and Casella [46] and modified by de los Campos et al. [47], and the BRR was performed as proposed by Pérez et al. [48]. Both the BRR and BL methods were implemented with BGLR software [44].

### Cross validation analysis

The set of 343 S_2:4_ lines was partitioned randomly into k = 3, 6 and 9 folds. For each combination of the prediction method, population parameter and phenotypic trait investigated, a model was calibrated using $\left[\frac{k-1}{k}\right]*343$ lines with genotypic and phenotypic data (229 lines for k = 3, 286 lines for k = 6 and 305 lines for k = 9), and the predictive capacity of the model was assessed by validating the estimated model with the lines in the left out fold (114 lines for k = 3, 57 lines for k = 6 and 38 lines for k = 9). The correlation between GEBVs predicted by the model and the observed phenotypic BLUP values was calculated. This process was repeated k-times so that each fold was left out once. The number of replicates (R) was chosen so that (k * R) ≥ 100 cross validation tests (i.e. R = 34 for k = 3, R = 17 for k = 6 and R = 12 for k = 9). Results of the same partitioning of lines into k-folds were used for the implementation of the three frequentist methods. New independent random partitioning was carried out for the two Bayesian methods for each of the 100 cross validation tests. Finally, the mean of the correlations (average accuracy) and the standard deviation across all cross validation tests were calculated for each k value.

The first cross validation experiment produced up to 55,296 correlations, resulting from 540 scenarios (four traits, five methods, three MAF, three LD and three k-fold values), with 100 to 108 tests for each scenario. These correlations (r) were then normalized, using a transformation function as proposed by Fisher [49] applying z = 0.5 * ln((1+r)/(1-r)). Once transformed, the correlations were analyzed as dependent variables in a variance analysis. ANOVA was performed to partition the variance into different sources, with all effects declared as fixed, and following three models: The simplest model compared all scenarios as a whole and gave the pooled dispersion in each scenario. The second model compared all the effects (trait, method, MAF, LD and k-fold ratio) without any interaction. The third model accounted for all effects as well as for all possible first-order interactions. The same ANOVA procedure was applied to analyze the other cross validation experiments that combined the 35 incidences matrices and the six population assignment methods.

## Results

### Phenotypic and genotypic characteristics of the population

#### Distribution and heritability of phenotypic traits

Of the four phenotypic traits investigated, only PH and PW were normally distributed. After log transformation of FL and YLD, variance analysis of all four traits revealed a highly significant effect for genotype (Table 1). Population structure (subpopulation) had a highly significant effect (*p* < 0.01) for PH and PW and a significant effect (*p* < 0.05) for FL and YLD. The replicate and block effects were highly significant for all traits. The results of ANOVA on the transformed variables were identical to those on non-transformed data. Consequently BLUP for individual S_2:4_ lines were calculated using untransformed data. Narrow sense heritability was high for FL and PH, moderate for YLD and low for PW (Table 1).

**Table 1:** Summary statistics of the four phenotypic traits, ANOVA results and heritability on phenotypes of the 343 S_2:4_ lines extracted from four subpopulations.

Statistics	Phenotypic traits			
	FL	PH	YLD	PW
Adjusted means (standard error)				
Subpopulation PCT4-C0 (n = 86)	79.59 (0.77)	90.16 (1.25)	37.21 (2.55)	2.09 (0.09)
Subpopulation PCT4-C1 (n = 83)	80.95 (0.79)	93.27 (1.27)	45.46 (2.57)	2.18 (0.09)
Subpopulation PCT4-C2 (n = 82)	78.06 (0.79)	99.34 (1.28)	45.09 (2.60)	2.39 (0.09)
Subpopulation PCT11-C1 (n = 92)	79.72 (0.75)	98.03 (1.22)	44.2 (2.49)	2.36 (0.09)
ANOVA results (*p*-values)				
Replicate	< 0.0001	< 0.0001	0.0003	0.0013
Subpopulation	0.0672	<0.0001	0.0156	0.0086
S_2:4_ lines (Subpopulation)	< 0.0001	< 0.0001	< 0.0001	0.0027
Variance components				
Block (Replicate)	1.59	12.85	96.04	0.13
S_2:4_ lines (Subpopulation)	46.03	89.89	214.55	0.12
Residual	4.16	34.74	273.68	0.61
Heritability (h^2^)	0.86	0.58	0.29	0.10

FL: days to flowering; PH: plant height; YLD: grain yield; PW: panicle weight; N and n: number of S_2:4_ lines that comprise the population and subpopulations, respectively. *p*-values from Fisher’s test to test the fixed effects.

**Fig 1:**
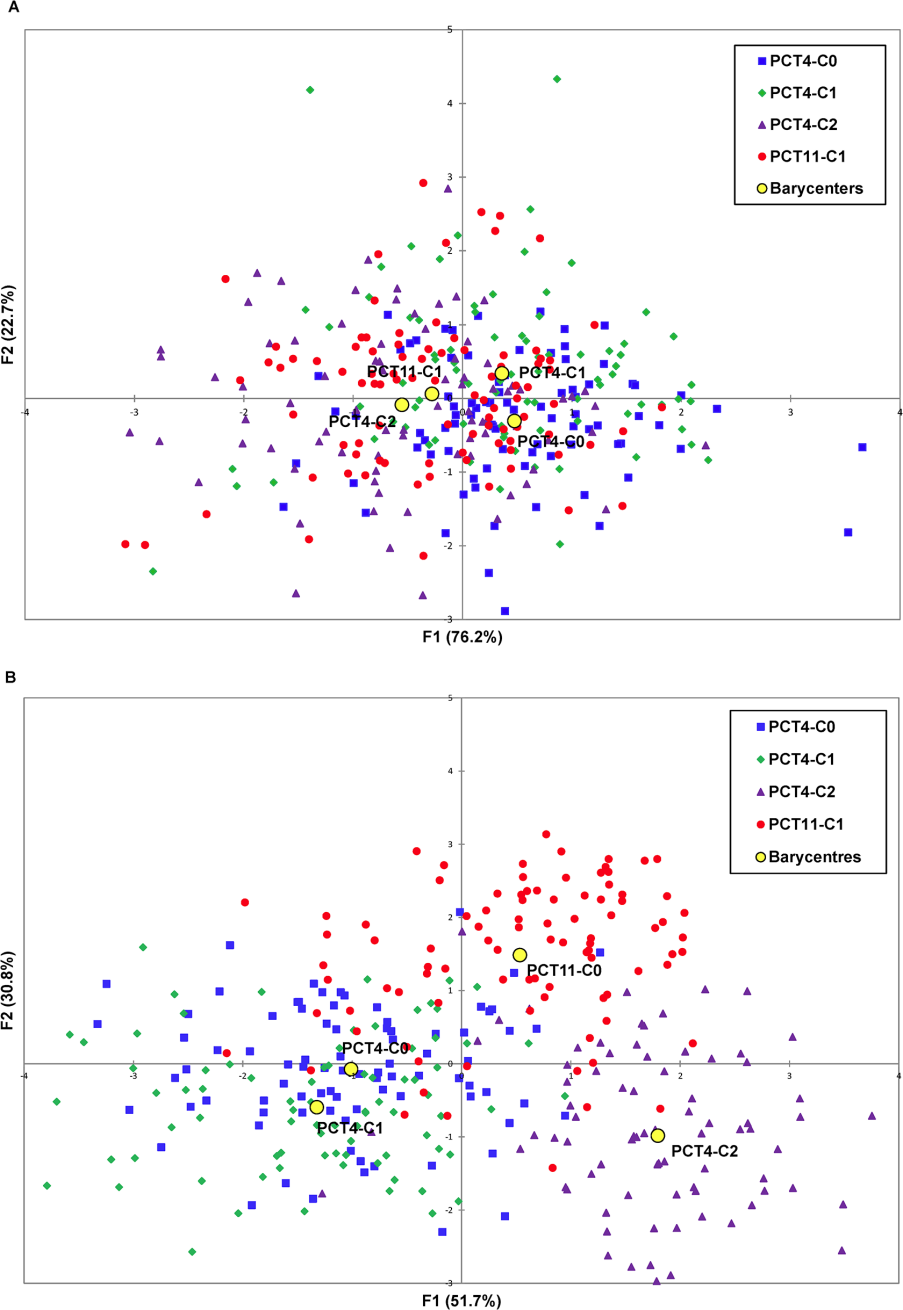
Projection of the 343 S_2:3_ lines on the first plane of a factorial discriminant analysis using (A) phenotypic data and (B) genotypic data.

Multidimensional analysis of phenotypic data confirmed the results obtained with ANOVA for the effect of subpopulation. Projection of the 343 S_2:4_ lines on the space defined by the two first axes of a FDA using the BLUP values for the four phenotypic traits (Fig 1A) showed a notable overlap between the four subpopulations. However, it also showed moderate differentiation when the barycenter of each subpopulation was considered. Fisher distances were highly significant (*p* < 0.001) between all pairs of subpopulations except PCT4-C2 and PCT11-C1 (Table 2).

**Table 2:** Pairwise Fisher distance (*F*_*D*_) and genetic differentiation (*F*_*ST*_) between subpopulations, effective population size (*Ne*) and number of monomorphic loci for each subpopulation (Nπ) out of 3,675 SNP.

	PCT4-C0	PCT4-C1	PCT4-C2	PCT11-C1	*Ne*	Nπ
PCT4-C0		4.526 **	11.568 ***	7.814 ***	32 ± 0.05	836
PCT4-C1	*0*.*025* *		10.422 ***	5.381 ***	28 ± 0.05	577
PCT4-C2	*0*.*045* *	*0*.*047* *		1.249 NS	48 ± 0.10	314
PCT11-C1	*0*.*050* *	*0*.*058* *	*0*.*045* *		53 ± 0.05	608

Pairwise *F*_*D*_ are presented above the diagonal and pairwise *F*_*ST*_ below the diagonal.

*, ** and ***: significant with *p* < 0.01, 0.001 and 0.0001, respectively; NS: non-significant.

#### Distribution of SNP markers

Across all chromosomes and all markers, the average marker density was one SNP every 44.8 kb, with densities ranging from 36.7 kb to 51.8 kb. The distribution of the 8,336 SNP loci along the 12 rice chromosomes is summarized in S1 Table and S1 Fig. The median (50% of the markers) for the distance between any pair of adjacent markers was 22.8 kb across chromosomes, ranging from 19.5 to 29.3 kb. For 88% of the pairs of adjacent markers, the distance was less than 100 kb, and in 22 cases, the distance was greater than 500 kb (data not shown).

#### Population characteristics

The distribution of allele frequencies at individual loci was markedly skewed toward unbalanced frequencies (S1 Table) and followed a beta law (S2 and S3 Figs) with mean β = 0.56, ranging from 0.38 to 0.68. The average MAF was 15.2% with a median of 9.6% and a third quartile of 23.7%. The proportion of loci with a MAF ≤ 10% was particularly high for chromosomes 4 and 9, 72% and 88%, respectively.

The average heterozygosity at the level of individual SNP markers was moderate (5.7%), with a median value of 4.8% and a maximum of 22%. The distribution of observed heterozygous loci (Ho) varied among the 12 rice chromosomes (S1 Table) and the median ranged from 2.8% to 6.5%. Among the 343 S_2:3_ lines, heterozygosity ranged from 0.6% to 32.4% with half the population accounting for 4.7% of heterozygous markers. At the scale of the entire genome, the distribution of Ho was right-skewed, with the highest fraction of loci within a class of 2–3% heterozygosity, and decreasing progressively until 20–21% heterozygosity, while the expected heterozygous loci (He = 2 p (1—p) / 4) shows a nearly U-shape distribution (S4 Fig). For all chromosomes Ho tended to be overestimated when the MAF was low and underestimated when the MAF was high (S4 Fig).

Effective population size *Ne* varied among the four subpopulations, from 28 for PCT4-C1 to 53 for PCT11-C1 (Table 2), reflecting the history of each subpopulation. The *Ne* calculated for the whole population of 343 S_2:3_ was 57.

#### Linkage disequilibrium

The LD, calculated for the population of 343 S_2:3_ lines was rather high and did not decrease rapidly with physical distance (S2 Table). The r^2^ values among the 12 chromosomes averaged 0.59 for marker pairs whose distance was between 0 and 25 kb, and ranged from 0.52 to 0.69. For all the chromosomes except chromosome 9, the r^2^ decreased to half its initial value when the distance between markers reached 500 to 650 kb. In the case of chromosome 9, the 50% decrease in r^2^ was reached at a distance of 1,500–2,000 kb between markers. The r^2^ values decreased to 0.2 at a distance of 900–1,500 kb for all chromosomes except chromosome 9. The average marker density of one SNP per 44.8 kb is thus enough for the estimation of the breeding value at the whole genome level.

#### Population structure

The projection of the 343 S_2:3_ lines on the space defined by the two first axes of the FDA (Fig 1B) revealed a complete overlap between PCT4-C0 and PCT4-C1, and a moderate overlap between these two subpopulations and PCT11-C1 and PCT4-C2. The heat map built from the kinship matrices (S5 Fig) confirmed the existence of a minor structure. Pairwise genetic differentiation evaluated by *F*_*ST*_ parameter was low but significant (*p* < 0.01) between all pairs of subpopulations (Table 2). Genotypic data had a higher power of discrimination than phenotypic data. As a result, the genotypic structure only partially overlapped the phenotypic structure. The existence of this genotypic structure provided a basis for evaluating the effect of the genetic distance between the training and validation populations on the accuracy of GEBVs.

### Accuracies of GEBV obtained by cross validation

In the first set of cross validation experiments, the 540 average accuracy (AA) values obtained differed greatly between traits, k-fold ratio, incidence matrices and regression methods (S3 Table). Overall, the highest AA was achieved for PH and the lowest AA for FL. For each trait, the optimal combination of factors varied. The highest AA was reached for PH (AA = 0.538) with BRR, k = 6, r^2^ ≤ 0.9 and MAF ≥ 5%, for PW (AA = 0.327) with BL, k = 9, r^2^ ≤ 1 and MAF ≥ 5%, for YLD (AA = 0.309) with BL, k = 9, r^2^ ≤ 0.9 and MAF ≥ 5%, and for FL (AA = 0.295) with LASSO, k = 6, r^2^ ≤ 0.9 and MAF ≥ 5% (Table 3).

**Table 3:** Best average accuracies among the 135 GEBVs obtained from the training data sets (TP) and the observed BLUP of the validation data sets (VP) considering each trait.

Trait	k-fold	LD (r^2^ ≤)	MAF (≥ %)	Number of SNP	Method	Average Accuracy
PH	6	0.9	5	4011	BRR	0.538 (0.082)
PW	9	1	5	5604	BL	0.327 (0.126)
YLD	9	0.9	5	4011	BL	0.309 (0.148)
FL	6	0.9	5	4011	LASSO	0.295 (0.113)

FL: days to flowering; PH: plant height; YLD: grain yield; PW: panicle weight; LD: linkage disequilibrium; MAF: minor allele frequency. Methods; LASSO: least absolute shrinkage and selection operator, BL: Bayesian LASSO, BRR: Bayesian ridge regression

#### Factors controlling the variation in prediction accuracy

For the first cross validation experiment, we used a factorial design to compare the 540 scenarios resulting from a combination of traits, methods, MAF, LD and k-fold ratio. This design made it possible to identify the effects of controlled factors and their interactions. Each single correlation between GEBVs and BLUP values was transformed as recommended by Fisher [49] to normalize the distribution so as to perform an ANOVA, whose results are presented in Table 4. Under the first model (539 degrees of freedom), ANOVA showed that the scenarios explained more than half of the total variation (R^2^ = 0.56) but that a high proportion of variation remained unexplained (CV = 39.6%). Under the second model, considering the main effects of controlled factors (13 degrees of freedom), ANOVA revealed very highly significant (*p* < 0.0001) effect of all factors, explaining 52% of total variation. The third ANOVA, which considered both main factors and all first-order interactions (79 degrees of freedom), showed that the interactions as a whole marginally improved the model (R^2^ = 0.55), although six of them proved to be very highly significant (*p* < 0.0001). Thus, when comparing R^2^ in model 1 and model 3, higher order interactions should explain even less variation.

**Table 4:** Sources of variation of the AA in the first cross validation experiment considering 540 scenarios.

	Model	Source	DF	SS	MS	F Value	Prob F		R^2^	CV	Root MSE
ANOVA fit statistics	1. Scenarios	Model	539	1092.21	2.03	127.14	< .0001	***	0.556	39.61	0.126
		Error	54625	870.60	0.02						
		Corrected Total	55164	1962.81							
	2. Individual factors	Model	13	1020.48	78.50	4594.18	< .0001	***	0.520	41.01	0.131
		Error	55151	942.33	0.02						
		Corrected Total	55164	1962.81							
	3. Factors & interactions	Model	79	1081.65	13.69	855.94	< .0001	***	0.551	39.68	0.126
		Error	55085	881.16	0.02						
		Corrected Total	55164	1962.81							
Test statistics for effects	Controlled factors^(1)^	Trait	3	987.38	329.13	19262.40	< .0001	***			
		Method	4	21.61	5.40	316.16	< .0001	***			
		k-fold	2	9.78	4.89	286.13	< .0001	***			
		LD	2	1.98	0.99	57.99	< .0001	***			
		MAF	2	0.89	0.45	26.11	< .0001	***			
	First-order interactions^(2)^	Method*Trait	12	52.27	4.36	272.28	< .0001	***			
		LD*Trait	6	4.24	0.71	44.14	< .0001	***			
		Trait*k-fold	6	1.99	0.33	20.77	< .0001	***			
		Method*LD	8	1.04	0.13	8.13	< .0001	***			
		Method*MAF	8	0.78	0.10	6.10	< .0001	***			
		Method*k-fold	8	0.61	0.08	4.77	< .0001	***			
		MAF*Trait	6	0.18	0.03	1.90	0.0766	NS			
		LD*MAF	4	0.02	0.00	0.28	0.8899	NS			
		MAF*k-fold	4	0.04	0.01	0.69	0.5999	NS			
		LD*k-fold	4	0.08	0.02	1.22	0.2984	NS			

Sources of variation were: method (BL, BRR, G-BLUP, RR-BLUP and LASSO), trait (FL, PH, YLD, PW), MAF (≥ 2.5, 5 and 10%), LD (≤ 0.75, 0.9, 1) and k-folds (k = 3, 6, 9).

^(1)^ The denominator term used was the mean square error (MSE) of model 2.

^(2)^ The denominator term used was the mean square error (MSE) of model 3.

#### Effect of the method of prediction

AA values differed significantly among the five methods of prediction implemented. The AA was the highest for BL (AA = 0.312) on average and lowest for LASSO (AA = 0.265) (Table 5). The AA obtained with BRR, G-BLUP and RR-BLUP were comparable and intermediate. The regression method interacted with each of the other factors (Table 4). The traits for which the highest differences were found across the five methods were FL, PW and YLD (S4 Table). For all traits except FL, the LASSO method led to the lowest AA. Since the performance of RR-BLUP was intermediate and because the process of calculation is comparatively rapid, only this method was used for the supplementary set of cross validation experiments.

**Table 5:** Adjusted means (LSMeans) of AA for controlled factors in the first cross validation experiment considering all 540 scenarios.

Controlled factor	Modality	LSMeans	¶
Method	BL	0.312	a
	BRR	0.307	b
	G-BLUP	0.304	b
	RR-BLUP	0.304	b
	LASSO	0.265	c
Trait	PH	0.489	a
	PW	0.274	b
	GW	0.239	c
	FL	0.192	d
k-fold ratio	9	0.308	a
	6	0.302	b
	3	0.285	c
LD (r^2^)	0.75	0.302	a
	0.90	0.301	a
	1	0.291	b
MAF	2.5	0.301	a
	5	0.301	a
	10	0.293	b

**¶** Different letters indicate significant differences (p < 0.05)

#### Effect of the k-fold ratio

**Fig 2:**
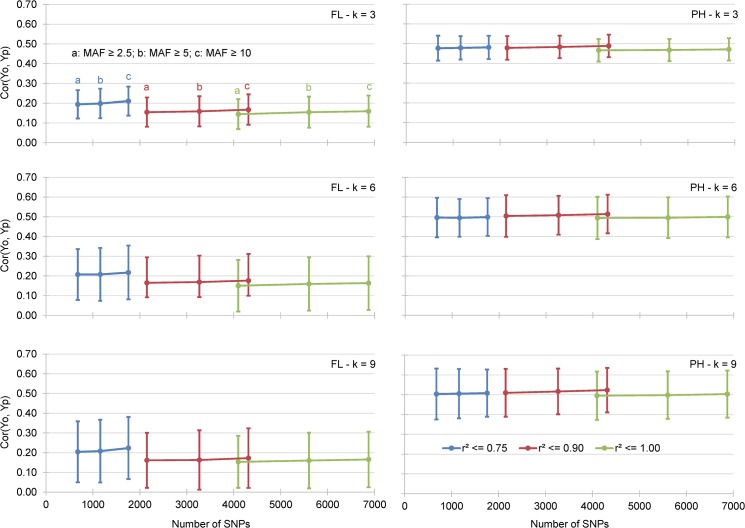
Mean correlation between GEBV obtained by cross validation of the training data set (Yp) and the observed BLUP values of the validation data sets (Yo). Results presented for 2 traits, 9 incidence matrices and 3 k-fold cross validation experiments.

The AA increased slightly with an increase in the k-fold ratio, AA = 0.285 for k = 3, AA = 0.302 for k = 6, and AA = 0.308 for k = 9 (Table 5). The differences were statistically significant, as shown by the average standard deviations for FL and PH, RR-BLUP prediction methods, k-fold cross validations (k = 3, 6 and 9) and the nine incidence matrices defined from the three MAF and the three r^2^ values (Fig 2). These differences are most likely due to the decrease in the size of the VP with an increase in the size of the TP, since in our cross validation experiments, these two sizes are mutually dependent. The standard deviation of the mean correlation, represented by the vertical bars, revealed an increase in AA that was associated with a significantly broader spread of the accuracies obtained for the 100 replicates of each cross validation experiment.

#### Effect of trait heritability on GEBV accuracy

**Fig 3:**
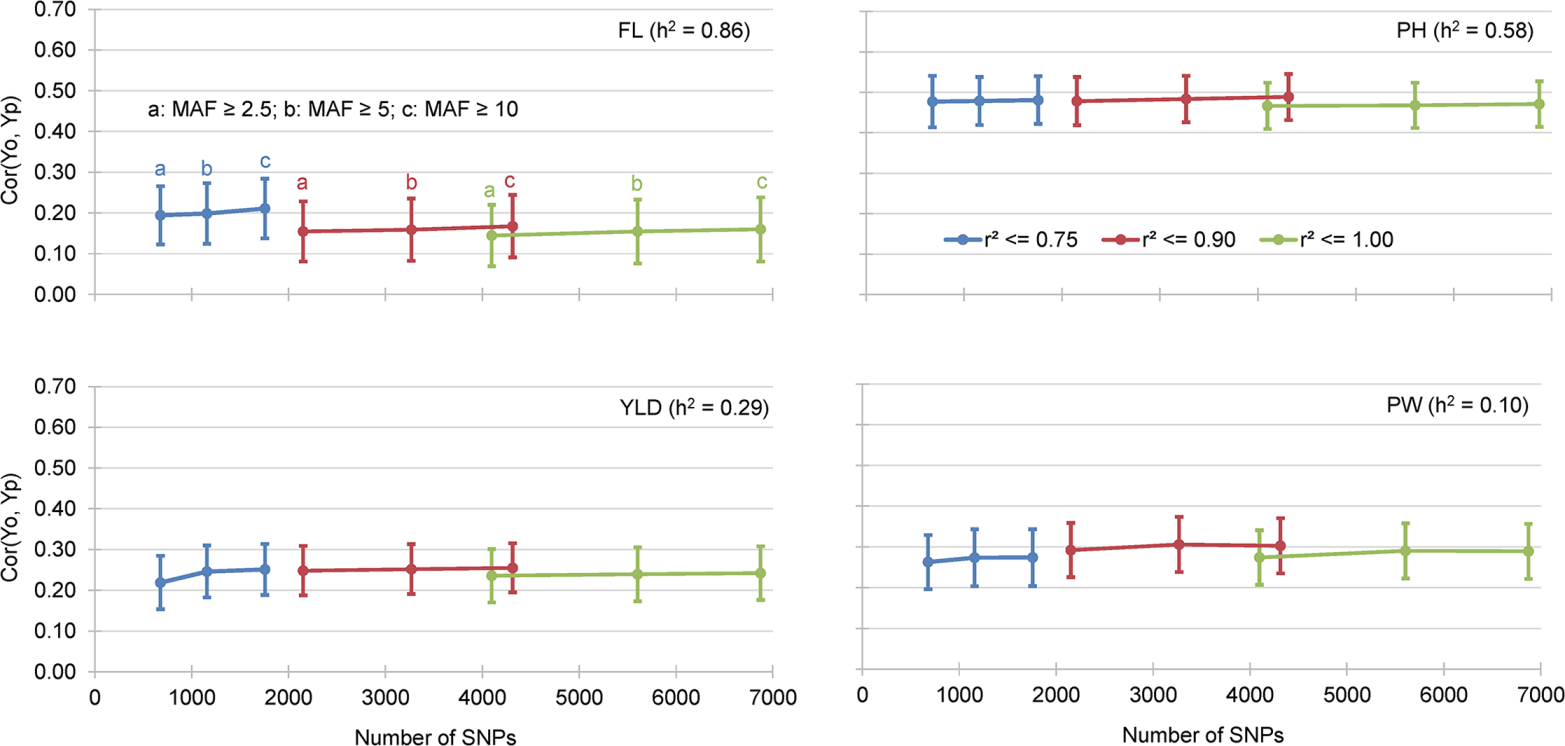
Mean correlation between GEBV obtained by cross validation of the training data set (Yp) and the observed BLUP values of the validation data sets (Yo). The results of 4 different traits and 9 incidence matrices are presented.

Highly significant (*p* < 0.0001) differences among AA were observed for the four phenotypic traits characterized by different narrow sense heritability (Table 5). Considering the RR-BLUP prediction methods with k = 3-fold cross validation and nine incidence matrices as previously defined, no simple relationship was observed between the AA and the level of trait heritability (Fig 3). The AA varied from AA = 0.192 for the trait with the highest heritability (FL, h^2^ = 0.86) to AA = 0.489 for the trait with the second highest heritability (PH, h^2^ = 0.58). YLD and PW had an intermediate level of AA (AA = 0.239 and AA = 0.274, respectively) despite some difference in heritability (0.29 and 0.10, respectively).

#### Effect of the size of the incidence matrix (marker density, LD and MAF)

**Fig 4:**
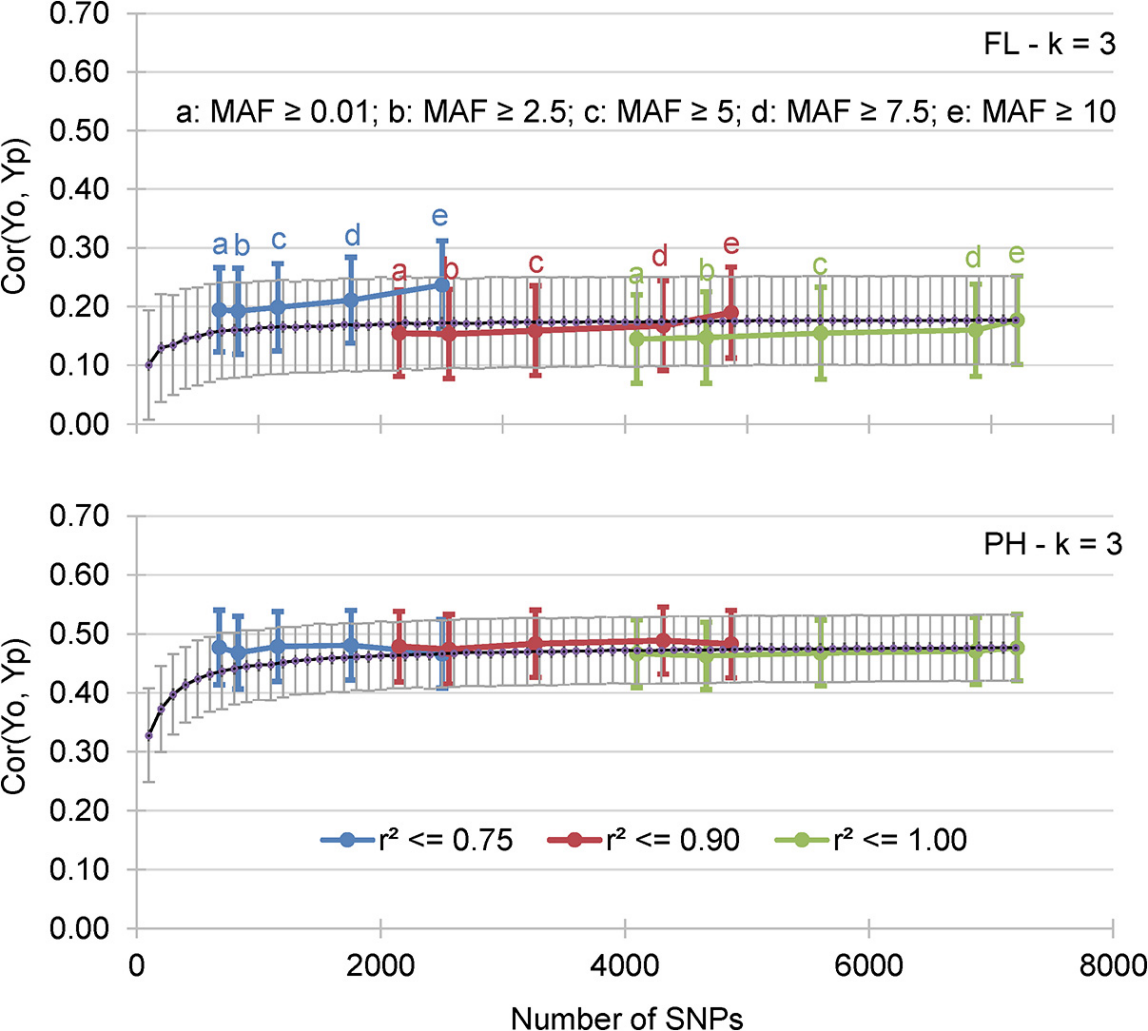
Mean correlation between GEBV obtained by cross validation of the training data set (Yp) and the observed BLUP values of the validation data sets (Yo). The results for flowering date (FL) and plant height (PH) and 15 incidence matrices are presented.

The AA among the 540 cross validation experiments decreased very slightly with an increase in the MAF threshold: AA = 0.301 for MAF ≥ 2.5%, AA = 0.301 for MAF ≥ 5% and AA = 0.293 for MAF ≥ 10% (Table 4). Likewise, accuracy increased slightly with a decrease in the LD threshold AA = 0.291 for r^2^ ≤ 1, AA = 0.301 for r^2^ ≤ 0.90 and AA = 0.302 for r^2^ ≤ 0.75. While MAF and LD had a significant effect on AA, their interaction did not significantly affect the accuracy of GEBVs (Table 4). However, a highly significant interaction (*p* < 0.0001) was detected between LD and phenotypic trait, which was not observed between MAF and traits, while both LD and MAF interacted significantly with the prediction methods. For all traits, prediction accuracy decreased significantly when no selection was made on LD (r^2^ ≤ 1) (S4 Table). For all methods except LASSO, AA was higher with lower LD and MAF thresholds (S4 Table). The non-significant interaction between MAF and LD on AA could be explained by the minor difference in the effects of each factor. To answer this question, we compared the accuracy observed using markers selected based on the combinations of five MAF thresholds (≥ 0.01, ≥ 2.5, ≥ 5, ≥ 7.5, and ≥ 10%) and three LD thresholds (r^2^ ≤ 0.75, ≤ 0.90 and ≤ 1), with the accuracy achieved using between 100 and 7,200 randomly sampled markers. This second set of cross validation experiments was performed with the RR-BLUP prediction model with k = 3-fold cross validation. Results for k = 3-fold cross validation and 15 incidence matrices resulting from the combination of five MAF thresholds and three LD thresholds are presented for FL and PH (Fig 4). For both traits and with randomly sampled markers, the AA first increased with the number of loci up to approximately 1,500 loci and then leveled off (Fig 4, black line). Marker selection based on combinations of r^2^ ≤ 0.75 with the five MAF thresholds covering an interval of 700 to 2,500 markers (Fig 4, blue line) always resulted in higher accuracy than random sampling of SNPs, and accuracy increased significantly with higher MAF values for FL. Slightly higher accuracy was achieved for any combinations of MAF thresholds with r^2^ ≤ 0.90 covering the interval of approximately 2,200 to 5,000 markers for PH (Fig 4, red line). However, the advantage of marker selection did not hold true for the combination of any MAF value with r^2^ ≤ 1 (Fig 4, green line), and in the case of FL, accuracy was slightly greater when the markers were selected randomly.

**Fig 5:**
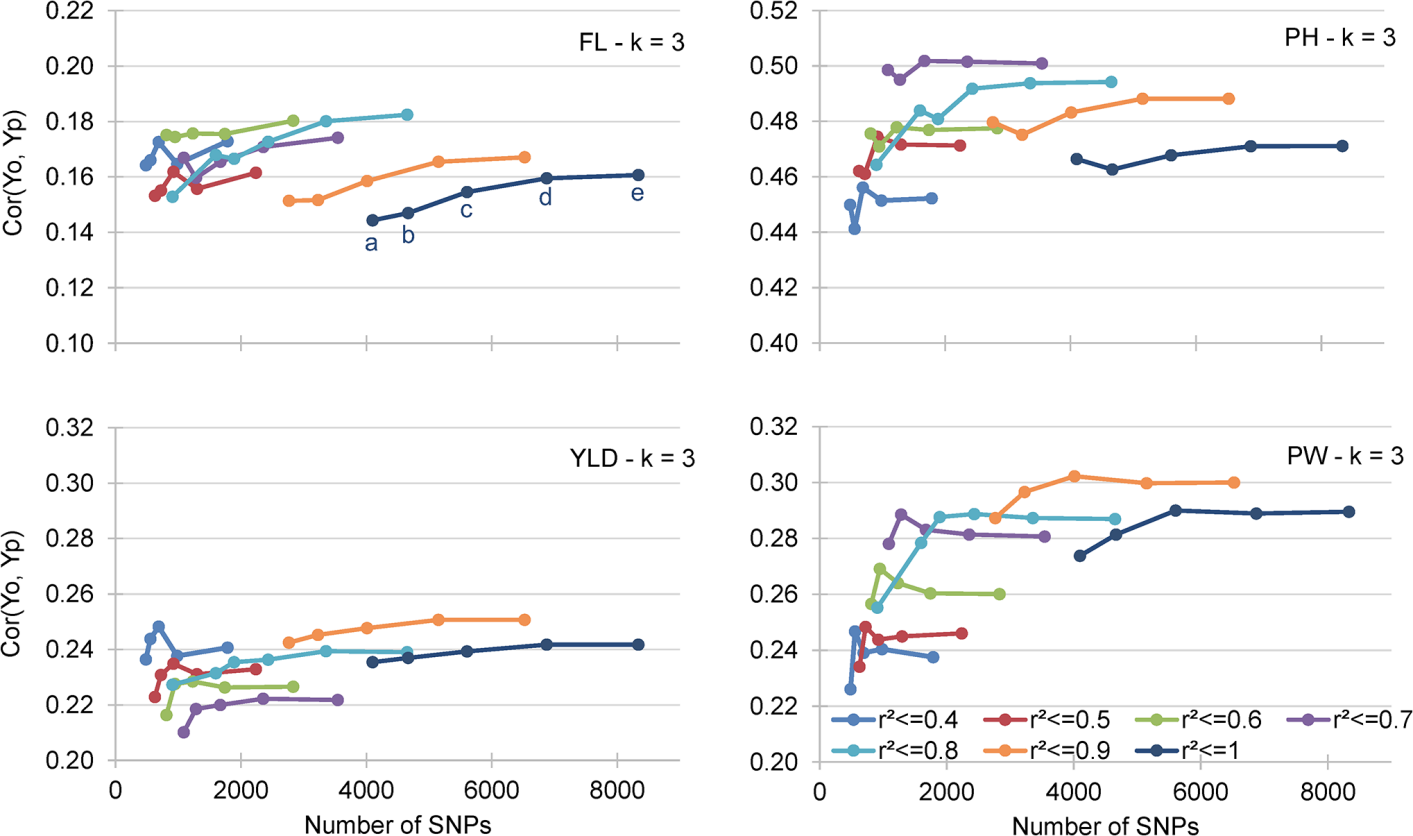
Mean correlation between GEBV obtained by cross validation of the training data set (Yp) and the observed BLUP values of the validation data sets (Yo). The results for days to flowering (FL), plant height (PH), panicle weight (PW) and grain yield (YLD) and 35 incidence matrices are presented.

These general trends were confirmed by a third set of 140 cross validation experiments with a broader range of LD threshold values (r^2^ ≤ 0.4 to r^2^ ≤ 1, with an increment of 0.1) tested under the RR-BLUP prediction model with k = 3-fold cross validation (S5 Table). Yet, when the different traits were considered separately for the 35 incidence matrices (Fig 5), the optimal thresholds of LD were reached with different levels of LD: r^2^ ≤ 0.6 for FL, r^2^ ≤ 0.7 for PH, and r^2^ ≤ 0.9 for YLD and PW (S5 Table). For all traits, variations in MAF threshold values <5% had little effect on accuracy, which started to decrease when MAF values were between 5 and 10% (S5 Table).

#### Kinship between the training and the validation population

**Fig 6:**
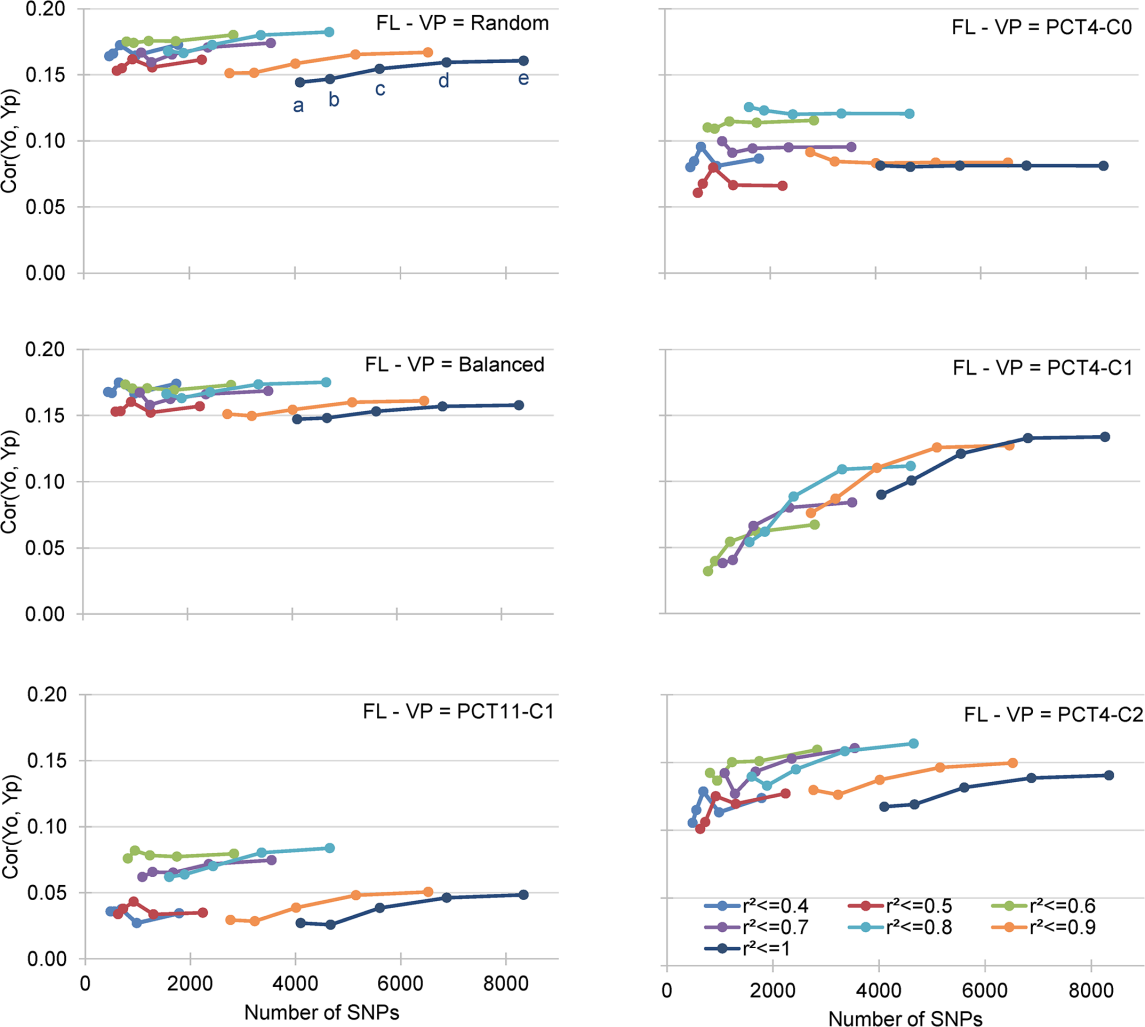
Mean correlation between GEBV obtained by cross validation of the training data set (Yp) and the observed BLUP values of the validation data sets (Yo). Results for day to flowering (FL) are presented for different composition of the validation population (VP).

In the 840 cross validation experiments performed to test the effect of kinship between the TP and VP, six different methods of sampling VP were tested (S6 Table). When the S_2:4_ lines comprising TP and VP were randomly sampled, without considering their membership of one of the four subpopulations, AA was 0.286, and the same results were obtained when the TP and VP represented a balanced share of each of the four subpopulations. The AA was significantly lower (AA = 0.231) when the TP comprised all the lines of three of the subpopulations and the VP comprised all the lines of the fourth subpopulation. In this sampling scenario, the lowest AA was observed when validation was performed using the PCT4-C0, PCT4-C1 or PCT4-C2 subpopulation, AA = 0.224, AA = 0.227 and AA = 0.231, respectively, and the AA was significantly higher (AA = 0.243) when PCT11-C1 was used as the VP (S6 Table). Significant interactions were observed between phenotypic traits and the sampling of TP when the RR-BLUP prediction method and nine incidence matrices were used (S6 Table, Fig 6 for FL, and S6–S8 Figs for the three other traits). The effect of the sampling strategy was highest for FL, AA = 0.165 with random sampling of VP, and AA = 0.052 when PCT4-C0, PCT4-C1 and PCT4-C2 were used to predict GEBVs in PCT11-C1. The least sensitive trait was PW, with a similar AA (0.223 to 0.285) for all the sampling strategies.

## Discussion

The aim of this study was to evaluate the potential of genomic selection to increase genetic gains in the context of rice population improvement through recurrent selection. The choice of this breeding scheme was based on the assumption that it is the most suitable for the improvement of complex quantitative traits, as it makes it possible to increase the frequency of favorable alleles while maintaining sufficient genetic variability for further crop improvement [34]. The expected increase in genetic gain is assumed to come from the acceleration of the RS process (more cycles per unit time), and from higher selection intensity, through genotypic evaluation of a larger number of candidates. Hereafter we (i) compare the accuracy of GEBV prediction to those reported in the literature (ii) discuss how the characteristics of our breeding population influenced the accuracy of genomic prediction, and (iii) under which conditions the promise of GS in increasing genetic gain can actually be fulfilled.

### Accuracy of GEBVs

The accuracy of genomic prediction in crop species obtained through cross validation using empirical data varies greatly. Most variation is attributed to the population structure and size, marker density and the genetic architecture of the traits. Generally, the accuracies of GEBV obtained in our study were lower than, or similar to, those reported in the rice literature. For instance, similar to our results, the accuracy of prediction for flowering in the *tropical japonica* component of a diversity panel of 413 accessions originating from 82 countries, ranged from 0.13 to 0.33. In the same experiment, the accuracy for plant height averaged 0.77 [15] versus 0.50 in our case. Likewise, the AA values observed for YLD and PW were similar to the accuracy reported for grain yield (0.304) in the rice *indica* population [17]. Conversely, for PH, the accuracy observed in that study (0.292) was much lower than in our study. Such a difference may reflect variability of heritability in the *tropical japonica* training population (h^2^ = 0.58 for PH) and in the *indica* population (h^2^ = 0.30 and 0.35 according to the season).

The results we obtained with the different regression methods differed significantly: the BL prediction model was slightly better (AA = 0.312), the BRR, G-BLUP and RR-BLUP methods resembled each other and intermediate, and the LASSO method produced lower AA values (AA = 0.265). Interactions between methods and traits were significant, mainly because LASSO produced a high AA for FL. The higher AA provided by LASSO compared to BLUP methods was the consequence of fitting a reduced number of explanatory variables to the model, setting some markers to exactly zero effect, thereby making it possible to avoid model over-parameterization for a trait controlled by a few major loci. As a matter of fact, looking at the effect of the markers for FL, in our population, a single locus with a strong effect was detected (data not shown). Similar results have been reported in the GS of fusiform rust resistance in loblolly pine, a trait controlled by a few genes with large effects [50] thus highlighting the importance of variable selection in GS. This result was also supported by the fact that selection of markers based on LD had the strongest effect on accuracy for FL. Similarly, Spindel reported greater accuracy of prediction for FL with the random forest algorithm, which is known to more effectively capture QTLs with large effect [17]. The difference between the AA obtained with LASSO and Bayesian LASSO was not expected, and was probably a consequence of differences in the sampling of S_2:4_ lines for TP and VP during the cross validation process.

### Effect of structure on the accuracy of genomic prediction

As expected, our synthetic population of 343 S_2:3_ lines exhibited a mild structure due to the breeding history of its four component subpopulations. GS was more accurate when the training set was more closely related to the selection candidates, i.e. when the TP and VP were made of a random set of all four subpopulations or from an equal proportion of each subpopulation. A similar effect of the composition of the TP has been reported in GS experiments using the rice diversity panel of 413 accessions. Accuracy within *indica* and *japonica* subpopulations was much greater than across the whole population due to a strong population structure accounting for 58.7% of overall molecular variance [16]. The authors applied different sampling algorithms to demonstrate that the optimization depends on the interaction between population structure and trait architecture where, for a polygenic trait the genome-wide relationship matrix may capture the phenotypic relationship, while for a non-polygenic trait, optimization may be achieved if the alleles controlling the trait are distributed according to the structure. In our study, considering all the 35 incidences matrices, the strongest effect of population structure was encountered for FL when one subpopulation was left out of the TP to serve as a VP, and the accuracy of the prediction were severely reduced when the three subpopulations together were used to predict FL in the PCT11-C1 subpopulation. The result of this un-stratified sampling suggests that the alleles controlling FL were not distributed according to the population structure, which was expected, as FL was not a criterion for subpopulation differentiation and varied among and within subpopulations. For our synthetic population, it would be interesting to analyze the accumulated length of shared haplotypes between the members of the TP and VP to assess the impact of LD relative to structure.

### Effect of LD on the accuracy of genomic prediction

The prequirement for high accuracy in GS is that the markers and QTLs are in strong linkage disequilibrium. When no prior information is available on the number and position of the QTLs, LD between SNPs can be used as a surrogate to evaluate the extent of LD in the population of interest. For quantitative traits controlled by many genes, in a multi-SNP effect detection approach, sampling the whole genome would ensure that the distribution of markers is in agreement with the LD of the population. In our population of 343 S_2:3_ lines, the short distance LD (r^2^ = 0.59 for distances of 0–25 kb) was similar to the one observed in a reference panel of 168 accessions representing the diversity of the tropical *japonica* group of *O*. *sativa* (to which our synthetic population belongs) genotyped in an identical GBS experiment [26]. However, the long distance LD (500–600 kb) decay was much slower than the one reported in the above mentioned reference panel and by other authors [26,51–53] in *japonica* backgrounds, which vary between 150 and 180 kb. Given these LD values, the marker density of 1 SNP per 45 kb we achieved is reasonably good coverage for the purpose of genomic GEBV prediction. In similar GS studies in diverse rice populations, the authors recommended the use of 6 and 7 k SNPs [17].

High long range LD can emerge from co-selection of favorable mutations in some regions of the genome conferring an adaptive advantage. However, in our case it was most probably due to the random associations of distant loci during the two-generation advance (S_0_ –S_2_) performed through single seed descent (SSD). High LD due to recent recombination events can reduce the accuracy of GEBV in cross validation experiments, as it is distributed within and not across the progenies. Likewise, it can rapidity reduce the accuracy of GEBV across breeding cycles, as it is highly susceptible to meiosis effects.

In our cross validation experiment, the accuracy of genomic prediction for PH, PW and YLD was affected by the choice of markers based on LD, and significantly higher accuracy was achieved for FL when the SNPs were selected with a LD threshold of r^2^ ≤ 0.75. The lower LD implies lower collinearity between markers, and collinearity was found to hamper the ability of regression methods to identify QTLs [54]. Indeed, we found significantly lower accuracies when all collinear markers were included (r^2^ ≤ 1). As suggested in other studies, the number of markers does not need to be high to achieve good genomic prediction, and best accuracy was achieved with no more than 7,142 well distributed SNPs (~ 1 SNP every 0.2 cM) in the irrigated rice population of breeding lines [17]. Based on simulation studies aimed at defining optimal marker density for GS, Solberg et al. [55] showed that accuracy reaches a plateau (> 0.8) at a marker density of 4*Ne*/morgan for an effective population size of 100 with 1,000 phenotypes and a heritability of 0.5. Understandably, this simulation depends on the genetic model of the trait concerned, and requires adjustment of the prior distribution to the true distribution of the QTL effects. In our case, with a map of 18 morgans (www.mapdisto.free.fr/), *Ne* ~ 50 implies that about 3,600 SNPs would be needed under an infinitesimal model with additive effects and under the assumption of evenly distributed QTLs on the chromosomes. For PH, PW and YLD, the greatest accuracies with the RR-BLUP method were achieved with matrix sizes ranging from 4,011 to 5,148 SNPs. Conversely, for FL, the greatest accuracies were achieved with a matrix size of 1,758 SNP, suggesting that the assumptions presented in the aforementioned simulation study do not apply to this trait. The low accuracy of the prediction of the highly heritable FL trait using the largest SNP matrices may result from high collinearity (the above mentioned long range LD) between markers and associated numerical instability.

### Effect of MAF on the accuracy of genomic prediction

Our interest in accounting for MAF in GS was motivated by the need to determine a reasonable MAF threshold to be used in the predictions. Our population of 343 S_2:3_ lines exhibited a number of polymorphic loci similar to the number observed in the reference tropical *japonica* panel [26] but the distribution of the MAF in those loci differed. Half the loci had an allelic frequency close to zero or one, leading to a U-shaped distribution. Likewise, several chromosomal regions had low MAF (≤ 11%) that were not reported in the reference panel. This MAF distribution may result from the allelic composition of the founder varieties of the synthetic populations or from the selection pressure to which the population has been subjected for more than 10 recombination cycles. Markers with low MAF contribute little to detectable phenotypic variation even when tightly linked with causal QTLs. On the other hand, even though those markers are statistically less informative in terms of the accuracy of individual predicted SNP values, they may be important for optimal representation of the genome and the relationships between individuals with methods such as G-BLUP. The results of our first cross validation experiment showed that the three MAF thresholds compared (≥ 2.5%, ≥ 5.0% and ≥ 10.0%) were differentiated by most methods. When a larger range of MAF was explored using the RR-BLUP method, the results revealed a complex relation between MAF and LD and the resulting marker density in determining accuracies. This relation varied with the traits concerned, suggesting a role for trait architecture in the relation between AA and MAF. At a given level of LD, considering markers with very low MAF did not improve prediction accuracy but significantly increased computation time (not shown). On the other hand, higher thresholds for MAF (≥ 5%) could negatively impact the accuracies, particularly if LD thresholds become too stringent, as a result of insufficient genome coverage. Scutari [56] showed that the penalized LASSO method retained both common and rare alleles when genotypes were first standardized to unit variance. In contrast, when a non-standardized incidence matrix was used in the model, the method preferentially retained common alleles and excluded rare alleles. Likewise, de los Campos et al. [34] showed that standardization caused stronger shrinkage for intermediate allele frequencies, and minimum shrinkage for extreme allele frequencies. Standardization thus appears to be suitable when the objective is to identify chromosomal segments that contribute to genotype-phenotype relations over the whole genome and independently of the current distribution of allele frequencies in the population. It is also suitable when the objective is the long-term improvement of the breeding population through a progressive increase in the frequency of favorable alleles. However standardization has also the drawback of promoting markers whose effect on phenotypic variation is not evaluated precisely. In the particular context of our RS-breeding program, it is not inconsequential to standardize the incidence matrix because (i) half the SNP markers have low allelic frequencies (MAF < 0.10) and (ii) rare and undetected favorable alleles are the basis for the recurrent breeding scheme, which aims to increase the frequency of such alleles in the population. For these reason, the best compromise may be (i) standardization after elimination of markers with the most unbalanced allele frequency and (ii) regular updating of the model across RS cycles to incorporate changes in allele frequencies. Finally, in forward-in-time simulations, mutation-drift equilibrium is an important issue as it acts as a stop criterion in the process of *in silico* simulation of base populations that are needed to help deal with high dimensional problems, to explore experimental designs for GS, or to test alternative hypotheses. Our synthetic population meets these conditions since the U-shaped distribution of MAF can be interpreted as a population in mutation-drift equilibrium [57].

### Conclusions

Here we evaluated the effect of different parameters (regression model, population structure, characteristics of the incidence matrix, and trait genetic architecture) on the accuracy of GEBV, in the context of rice population improvement through recurrent selection. Although a rather low level of accuracy of GEBVs was observed in any combination of levels of the different parameters, this first study does not call into question the potential of genomic selection (GS) to increase genetic gains in the context of this breeding scheme. First, because the observed accuracy of GEBV was of the same magnitude as the heritability of complex traits such as grain yield, and GS can potentially accelerate genetic gain by increasing selection intensity (provided genotyping of a large number of entries is possible) and by shortening the selection cycle with genomic predictions approximating future pure lines performances in an earlier generation (S_1_ instead of S_2_). Second, the accuracy of GEBV can be further improved through more favourable germplasm development options, such as switching from single seed descent to a bulk method when advancing from generation S_0_ to S_2_. To further explore the potential of GS, we are currently developing a more analytical approach using simulation tools for the development of the training population.

## Supporting Information

S1 Fig Distribution of SNP markers on the 12 chromosomes according to their physical position after imputation of missing data. (TIF)

S2 Fig Distribution of allele frequency observed at the 8,336 marker loci along the 12 chromosomes after imputation of missing data. The U shape can be approximated by a beta distribution with shape parameters (alpha, beta) = (0.56, 0.56).
(TIF)

S3 Fig Distribution of frequencies of minor alleles (MAF) along the 12 chromosomes observed at 8,336 SNP loci after imputation of missing data. (TIF)

S4 Fig Distribution of observed (Ho) and expected (He) frequencies of heterozygotes (1), and their relation with MAF over 8,336 SNP loci after imputation of missing data (2). (TIF)

S5 Fig Heat map representing pairwise kinship between the 343 S_2:3_ lines using 8,336 SNP loci after imputation of missing data. (TIF)

S6 Fig Variation in the prediction accuracy of plant height (PH) according to the composition of the training (TP) and validation (TV) populations. The prediction method was RR-BLUP with k = 3-fold cross validation; r^2^: linkage disequilibrium; a, b, c, d and e: minor allele frequency (MAF) thresholds of ≥ 0%, ≥ 2.5%, ≥ 5%, ≥ 7.5% and ≥ 10%.
(TIFF)

S7 Fig Variation in the prediction accuracy of gain yield (YLD) according to the composition of the training (TP) and validation (TV) populations. The prediction method was RR-BLUP with k = 3-fold cross validation; r^2^: linkage disequilibrium; a, b, c, d and e: minor allele frequency (MAF) thresholds of ≥ 0%, ≥ 2.5%, ≥ 5%, ≥ 7.5% and ≥ 10%.
(TIFF)

S8 Fig Variation in the prediction accuracy of panicle weight (PW) according to the composition of the training (TP) and validation (TV) populations. The prediction method was RR-BLUP with k = 3-fold cross validation; r^2^: linkage disequilibrium; a, b, c, d and e: minor allele frequency (MAF) thresholds of ≥ 0%, ≥ 2.5%, ≥ 5%, ≥ 7.5% and ≥ 10%.
(TIFF)

S1 Table Summary information on the distribution of the 8,336 SNP loci, distribution of the minor allele frequencies (MAF), and distribution of heterozygosity along the 12 rice chromosomes. (XLSX)

S2 Table Average linkage disequilibrium (r^2^) between marker pairs according to chromosomes and the distance between markers, considering loci with MAF ≥ 2.5%. (XLSX)

S3 Table Average accuracies between 108 GEBVs obtained for the training data sets (TP) and the observed BLUP of the validation data sets (VP) considering three k-fold ratios between TP and VP. Mean of correlations (standard deviation) across the 100 cross validation replicates
(XLSX)

S4 Table Least square means (LSMeans) for first set of 540 cross validation experiments to test the significant difference between grouped effects; methods x trait, methods x k-fold, methods x LD, methods x MAF. Different letters indicate significantly different least square means LSMeans.
(XLSX)

S5 Table Least square means (LSMeans) for the second set of 140 cross validation experiments including 35 incidence matrices and tests of the significant difference between grouped effects; LD x trait, MAF x traits. Different letters indicate significantly different LSMeans.
(XLSX)

S6 Table Least square means (LSMeans) for the third set of 840 cross validation experiments testing the significant difference between grouped effect of different scenarios for the sampling of training and validation populations. (XLSX)
