# Correction: Accuracy of Genomic Selection in a Rice Synthetic Population Developed for Recurrent Selection Breeding

**DOI:** 10.1371/journal.pone.0154976

**Published:** 2016-05-19

**Authors:** Cécile Grenier, Tuong-Vi Cao, Yolima Ospina, Constanza Quintero, Marc Henri Châtel, Joe Tohme, Brigitte Courtois, Nourollah Ahmadi

Figs 2, 3 and 4 are incorrect. Additionally, there are errors in the legends for Figs 5 and 6, as well as S6 Fig, S7 Fig, and S8 Fig.

Please see the corrected Fig 2, Fig 3 and Fig 4 here. Additionally, please find the legends for Fig 2, Fig 3, Fig 4, Fig. 5, Fig. 6, S6 Fig, S7 Fig, and S8 Fig below.

## Figure Legends

**Fig 2:**
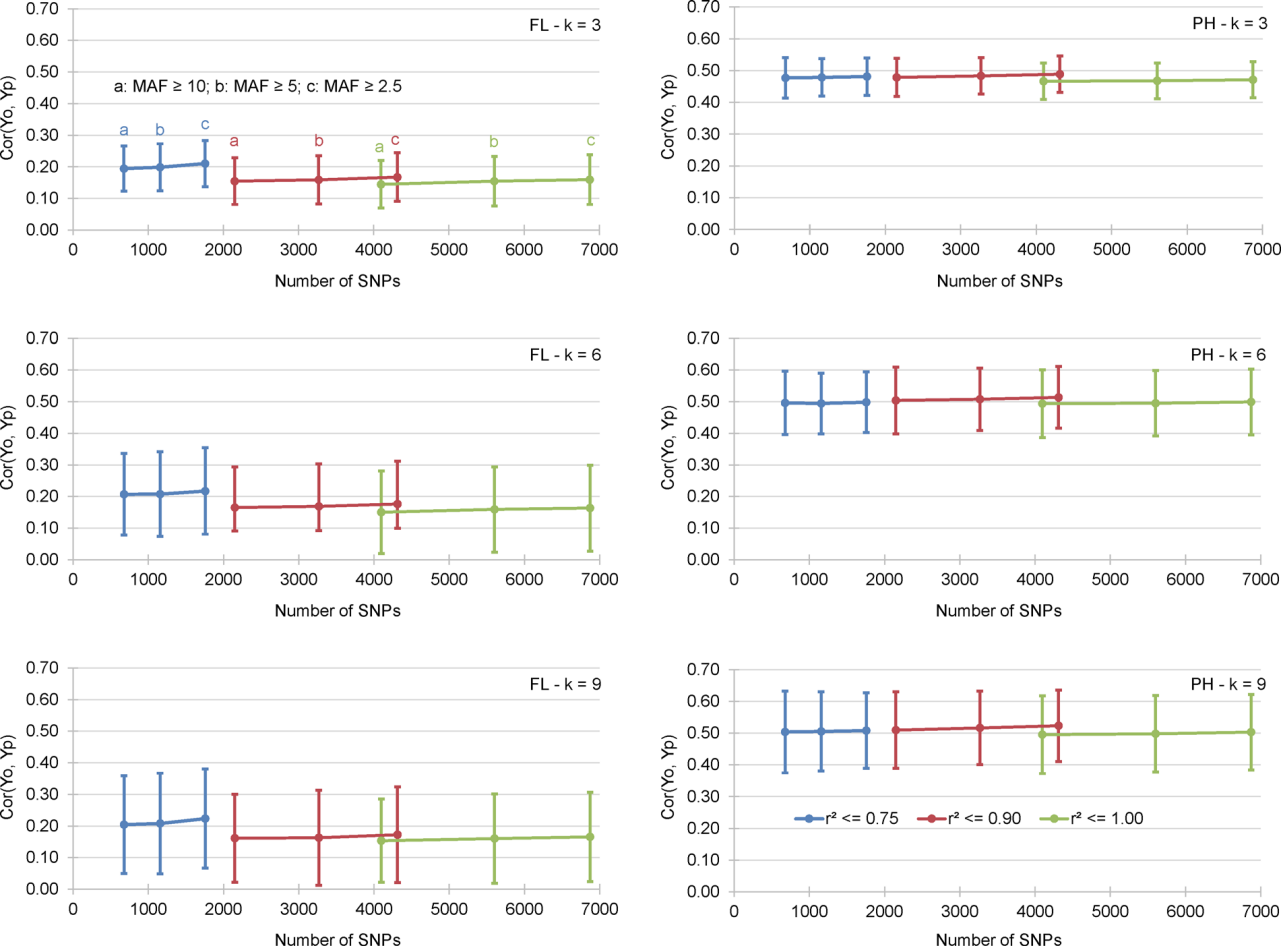
Mean correlation between GEBV obtained by cross validation of the training data set (Yp) and the observed BLUP values of the validation data sets (Yo). Results presented for 2 traits, 9 incidence matrices and 3 k-fold cross validation experiments.

**Fig 3:**
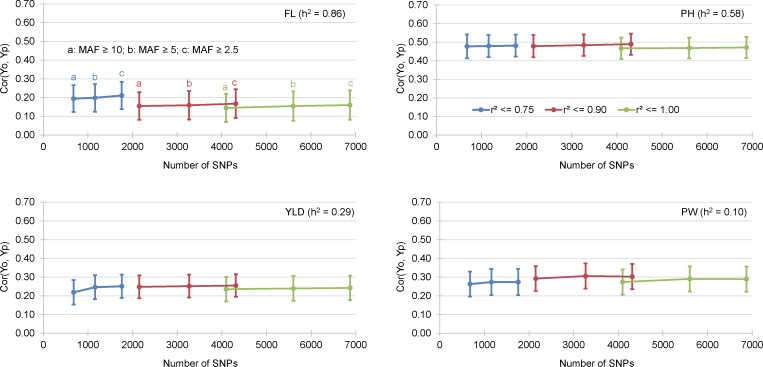
Mean correlation between GEBV obtained by cross validation of the training data set (Yp) and the observed BLUP values of the validation data sets (Yo). The results of 4 different traits and 9 incidence matrices are presented.

**Fig 4:**
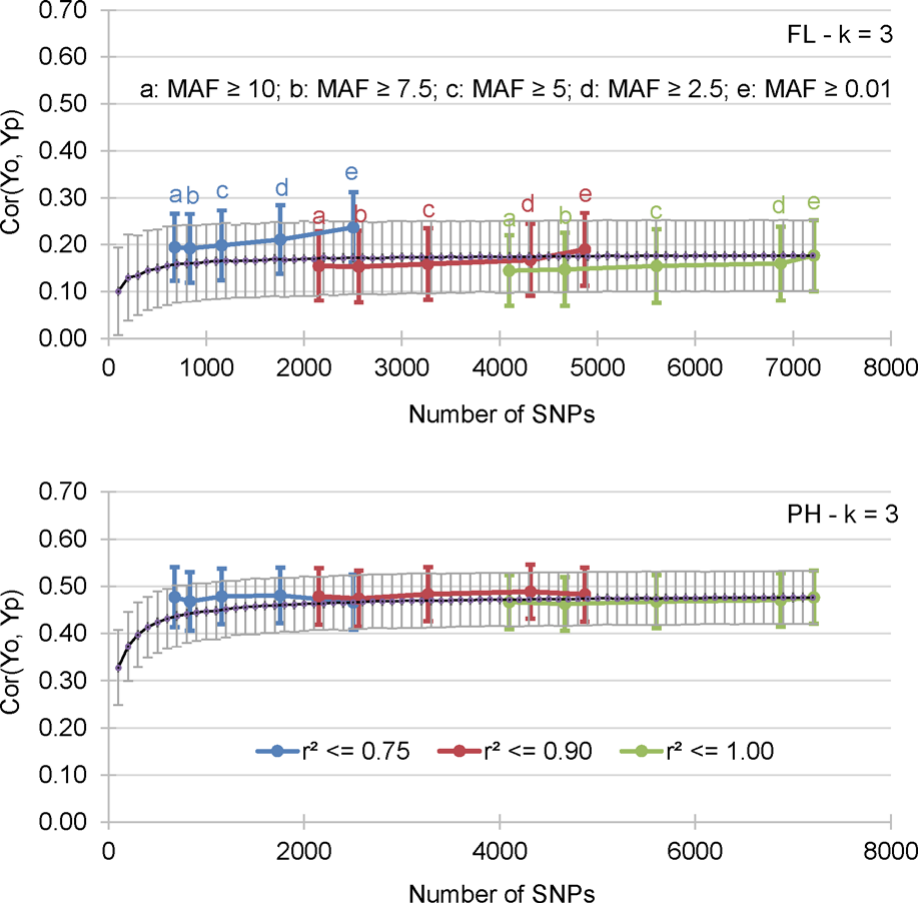
Mean correlation between GEBV obtained by cross validation of the training data set (Yp) and the observed BLUP values of the validation data sets (Yo). The results for flowering date (FL) and plant height (PH) and 15 incidence matrices are presented.

**Fig 5**: **Mean correlation between GEBV obtained by cross validation of the training data set (Yp) and the observed BLUP values of the validation data sets (Yo).** The results for days to flowering (FL), plant height (PH), panicle weight (PW) and grain yield (YLD) and 35 incidence matrices are presented (a, b, c, d and e: minor allele frequency (MAF) thresholds of ≥ 10%, ≥ 7.5%, ≥ 5%, ≥ 2.5% and ≥ 0.01%).

**Fig 6**. **Mean correlation between GEBV obtained by cross validation of the training data set (Yp) and the observed BLUP values of the validation data sets (Yo).** Results for day to flowering (FL) are presented for different composition of the validation population (VP) and 35 incidence matrices (a, b, c, d and e: minor allele frequency (MAF) thresholds of ≥ 10%, ≥ 7.5%, ≥ 5%, ≥ 2.5% and ≥ 0.01%).

## Supporting Information Legends

**S6 Fig.**: **Variation in the prediction accuracy of plant height (PH) according to the composition of the training (TP) and validation (TV) populations**. The prediction method was RR-BLUP with k = 3-fold cross validation; r²: linkage disequilibrium; a, b, c, d and e: minor allele frequency (MAF) thresholds of ≥ 10%, ≥ 7.5%, ≥ 5%, ≥ 2.5% and ≥ 0.01%.

**S7 Fig.**: **Variation in the prediction accuracy of gain yield (YLD) according to the composition of the training (TP) and validation (TV) populations**. The prediction method was RR-BLUP with k = 3-fold cross validation; r²: linkage disequilibrium; a, b, c, d and e: minor allele frequency (MAF) thresholds of ≥ 10%, ≥ 7.5%, ≥ 5%, ≥ 2.5% and ≥ 0.01%.

**S8 Fig.**: **Variation in the prediction accuracy of panicle weight (PW) according to the composition of the training (TP) and validation (TV) populations**. The prediction method was RR-BLUP with k = 3-fold cross validation; r²: linkage disequilibrium; a, b, c, d and e: minor allele frequency (MAF) thresholds of ≥ 10%, ≥ 7.5%, ≥ 5%, ≥ 27.5% and ≥ 0.01%.
