# Supplementary figures and images for: Accuracy of Genomic Selection in a Rice Synthetic Population Developed for Recurrent Selection Breeding
Source: PLoS One. 2015 Aug 27;10(8):e0136594. doi: 10.1371/journal.pone.0136594 (PMC4551487; doi:10.1371/journal.pone.0136594)

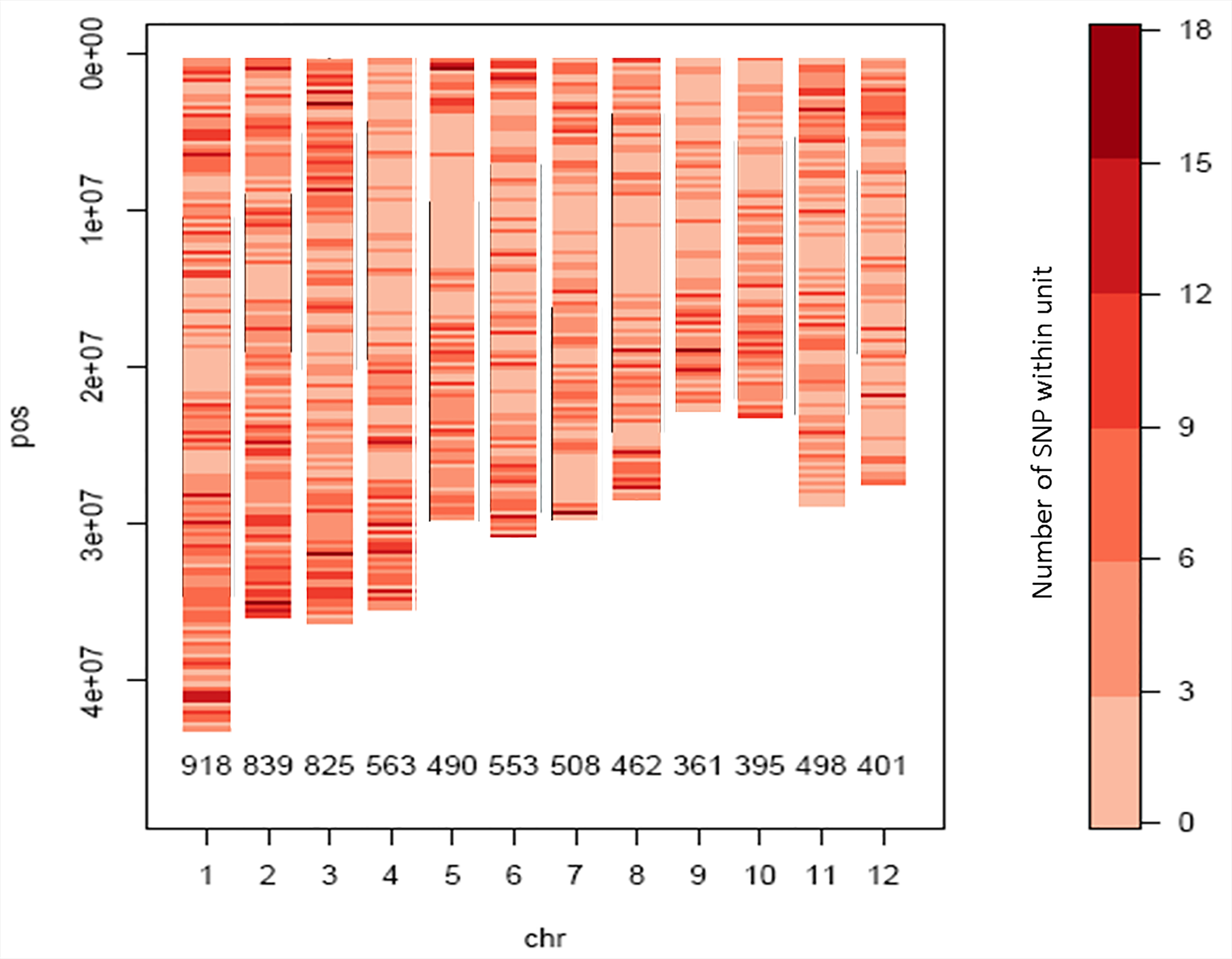

Supplement: S1 Fig — (TIF) [file pone.0136594.s001.tif]

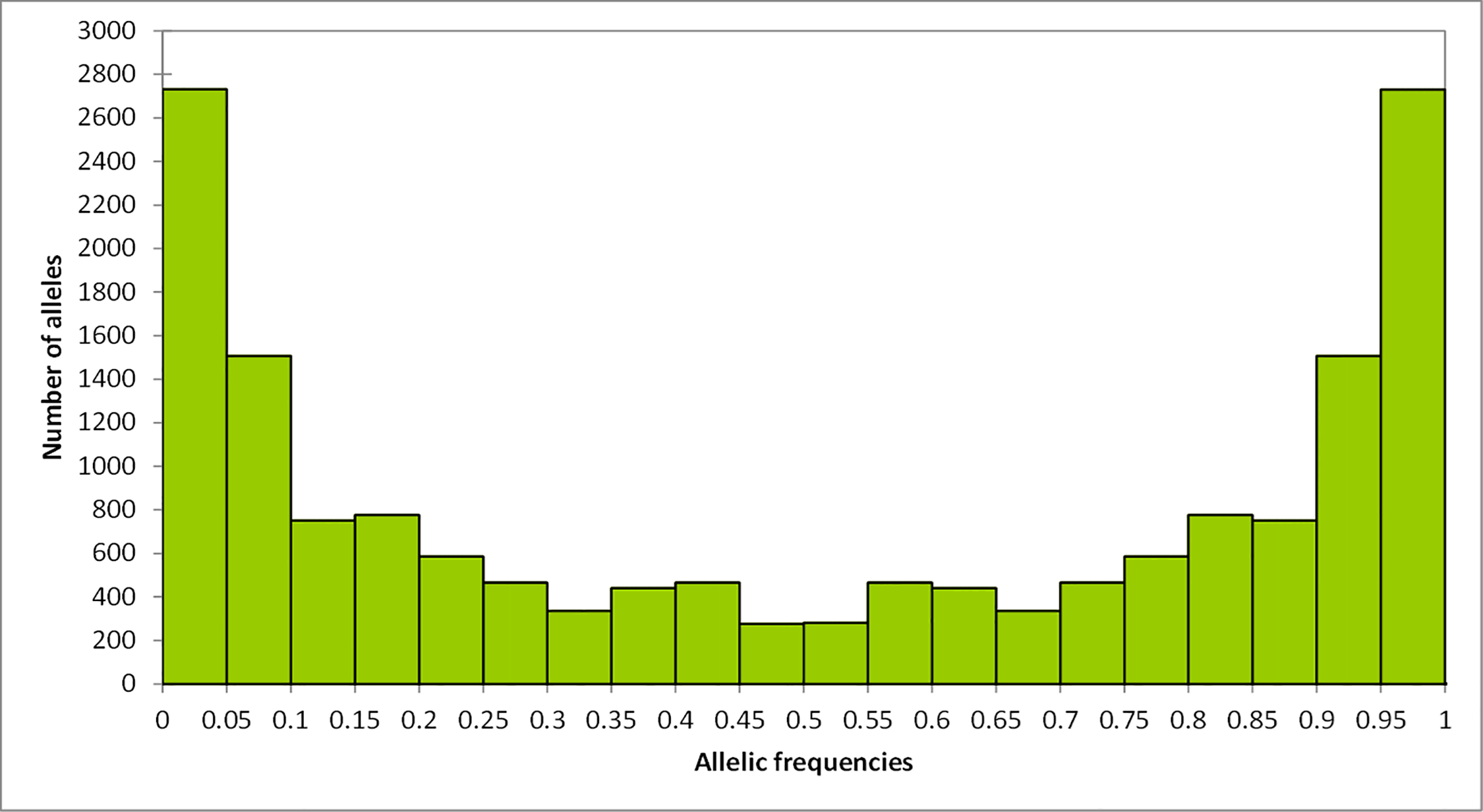

Supplement: S2 Fig — The U shape can be approximated by a beta distribution with shape parameters (alpha, beta) = (0.56, 0.56). (TIF) [file pone.0136594.s002.tif]

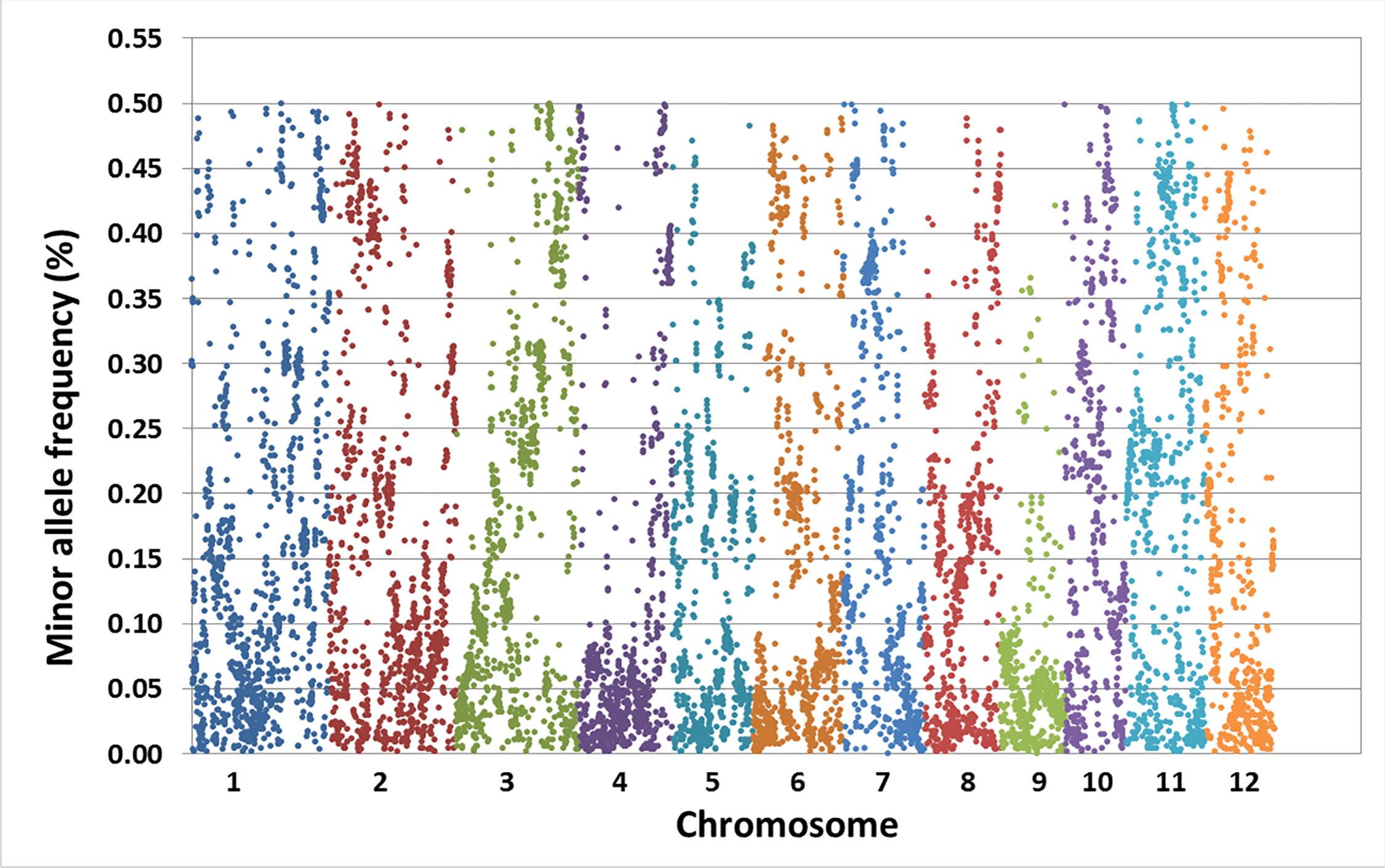

Supplement: S3 Fig — (TIF) [file pone.0136594.s003.tif]

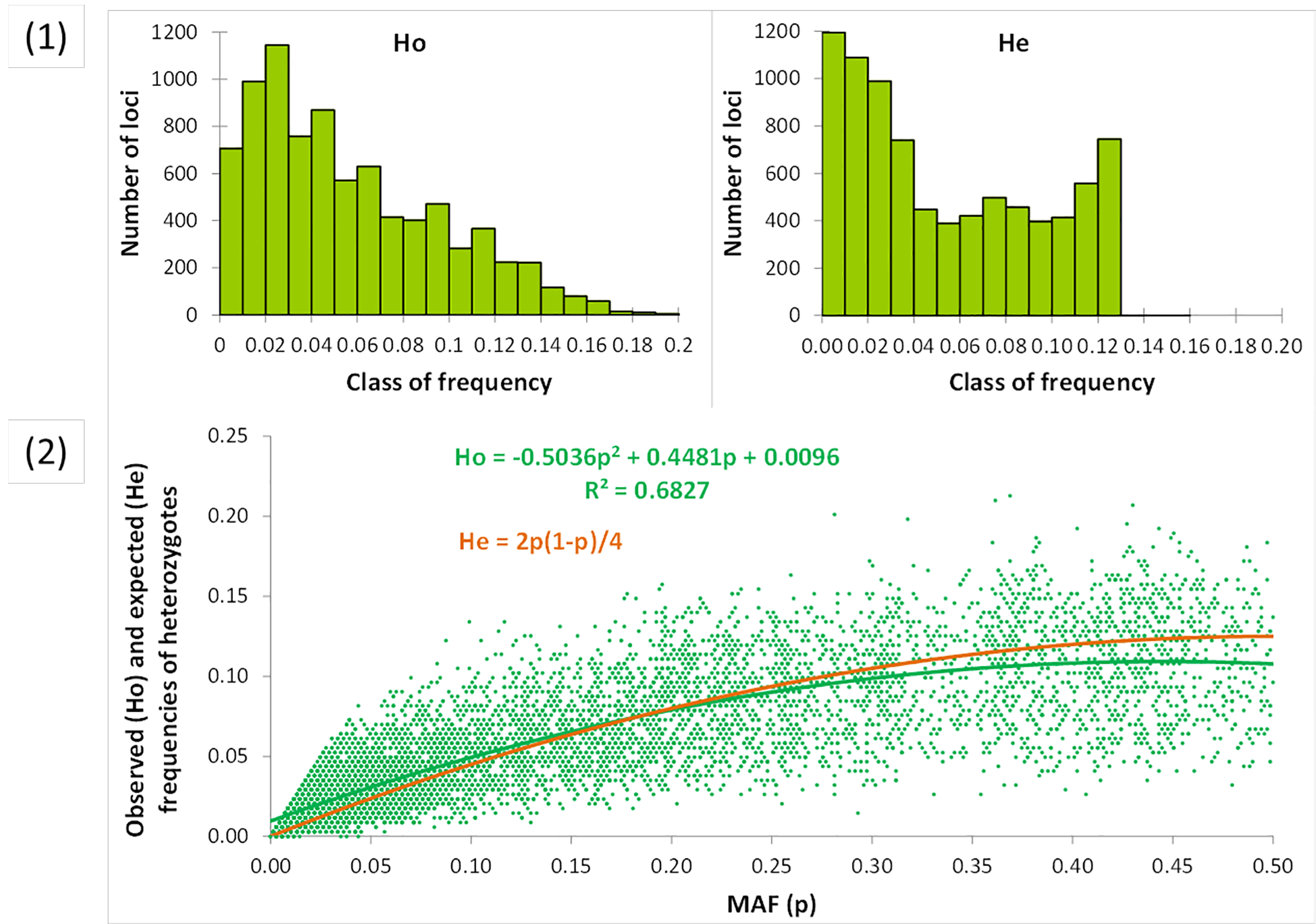

Supplement: S4 Fig — (TIF) [file pone.0136594.s004.tif]

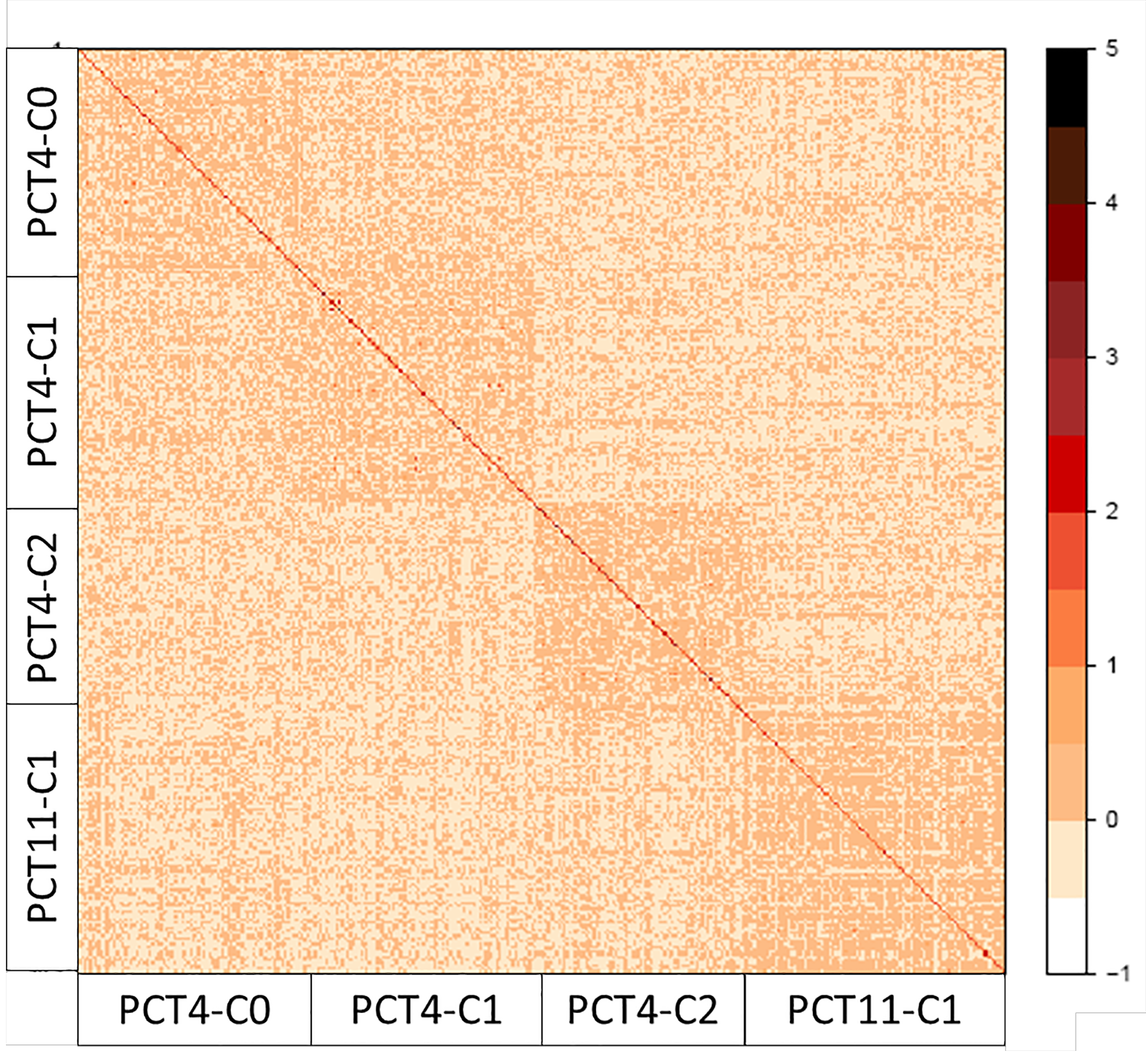

Supplement: S5 Fig — (TIF) [file pone.0136594.s005.tif]

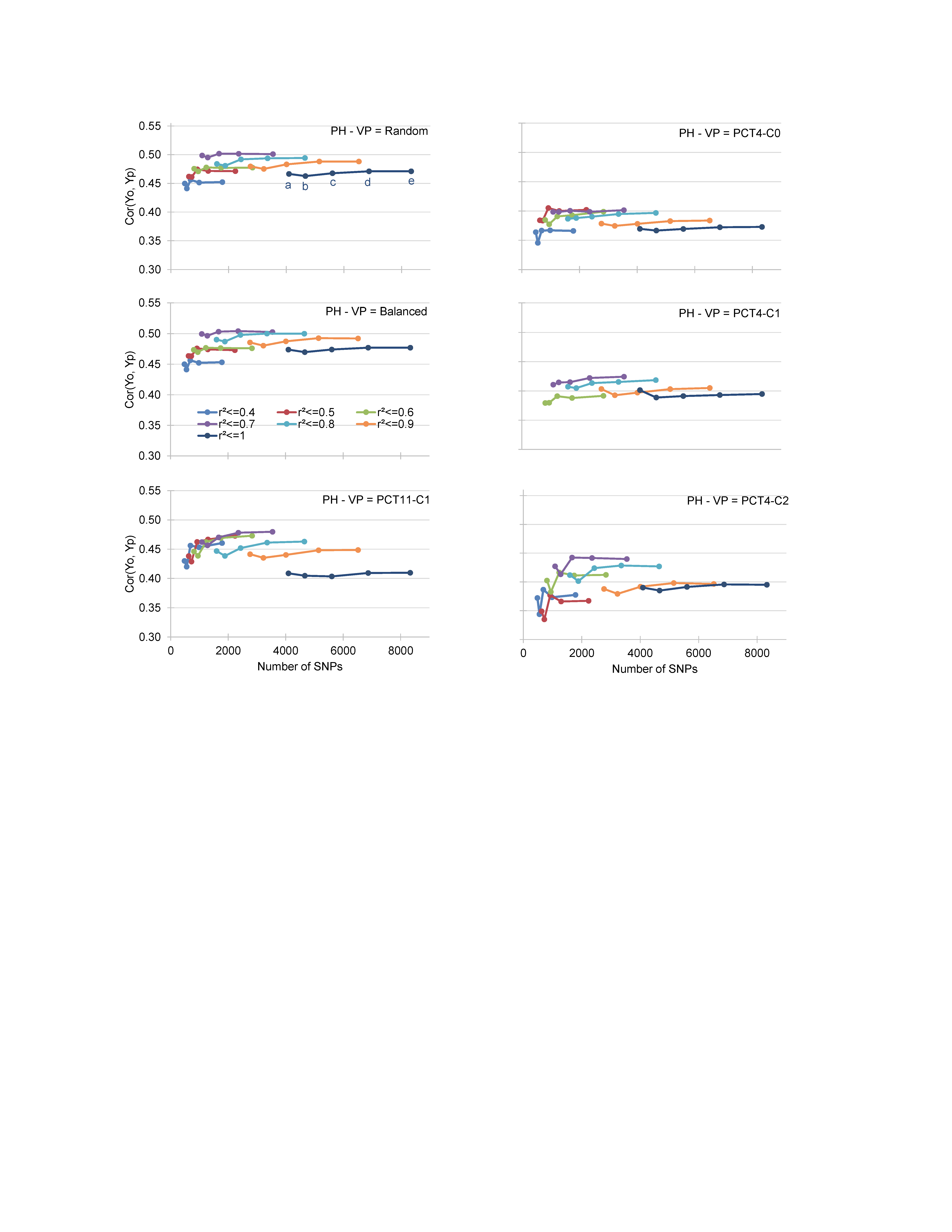

Supplement: S6 Fig — The prediction method was RR-BLUP with k = 3-fold cross validation; r2: linkage disequilibrium; a, b, c, d and e: minor allele frequency (MAF) thresholds of ≥ 0%, ≥ 2.5%, ≥ 5%, ≥ 7.5% and ≥ 10%. (TIFF) [file pone.0136594.s006.tiff]

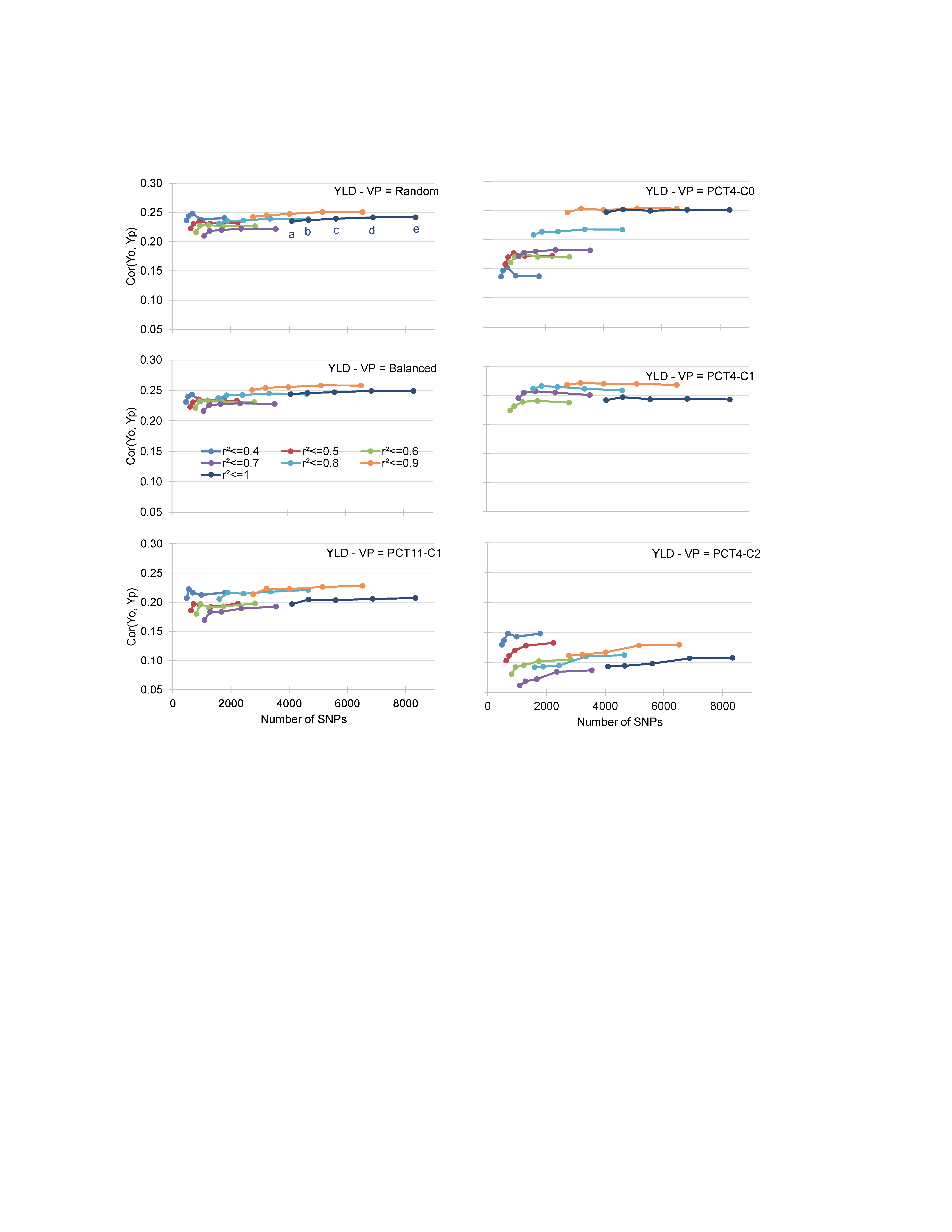

Supplement: S7 Fig — The prediction method was RR-BLUP with k = 3-fold cross validation; r2: linkage disequilibrium; a, b, c, d and e: minor allele frequency (MAF) thresholds of ≥ 0%, ≥ 2.5%, ≥ 5%, ≥ 7.5% and ≥ 10%. (TIFF) [file pone.0136594.s007.tiff]

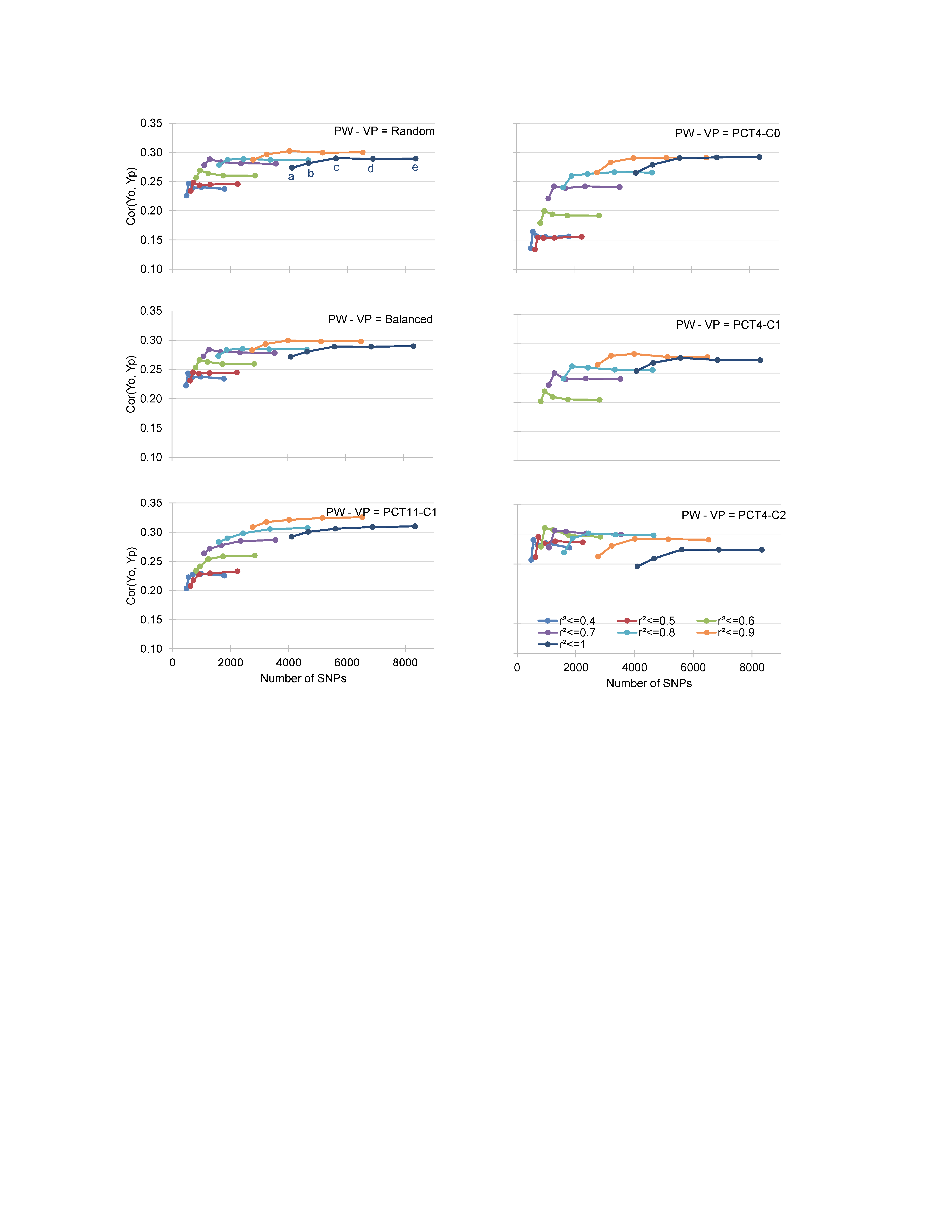

Supplement: S8 Fig — The prediction method was RR-BLUP with k = 3-fold cross validation; r2: linkage disequilibrium; a, b, c, d and e: minor allele frequency (MAF) thresholds of ≥ 0%, ≥ 2.5%, ≥ 5%, ≥ 7.5% and ≥ 10%. (TIFF) [file pone.0136594.s008.tiff]
